# Sensors in Self-Driving Vehicles: A Detailed Literature Review and New Trends

**DOI:** 10.3390/s26072153

**Published:** 2026-03-31

**Authors:** Patrik Viktor, Gabor Kiss

**Affiliations:** 1 Keleti Károly Faculty of Business and Management, Obuda University, 1034 Budapest, Hungary; viktor.patrik@uni-obuda.hu; 2 Institute of Safety Science and Cybersecurity, Obuda University, 1034 Budapest, Hungary

**Keywords:** autonomous vehicles, sensor systems, LiDAR, radar, camera, ultrasonic sensors, event camera, thermal camera, IMU, GNSS, V2X communication, FMCW LiDAR, terahertz radar, functional safety

## Abstract

Autonomous vehicles rely on complex sensing systems to perceive their environment and ensure safe operation. This review analyses the main sensor technologies used in self-driving vehicles, including cameras, LiDAR, radar, ultrasonic sensors and GNSS/IMU-based localisation systems. A core set of 40 primary research articles was systematically analysed to compare the capabilities, limitations and integration challenges of sensing technologies used in autonomous vehicles. In addition to these primary studies, further references were included to provide background information and describe emerging developments in autonomous sensing systems. The review shows that no single sensor technology can provide reliable perception under all environmental conditions. Camera systems offer rich visual information but are sensitive to lighting and weather conditions, while LiDAR provides highly accurate three-dimensional geometry but suffers from signal attenuation in rain and fog. Radar sensors demonstrate superior robustness in adverse weather and enable direct velocity measurement, although their spatial resolution remains limited compared to optical sensors. As a result, modern autonomous vehicles rely on multi-sensor fusion architectures that combine complementary sensing modalities to improve reliability and safety. The analysis also identifies several key research gaps in the current literature. In particular, there is a lack of systematic evaluation of trade-offs between sensor performance, computational requirements and vehicle energy consumption. Furthermore, the safety certification of artificial intelligence-based perception systems and the integration of emerging technologies such as FMCW LiDAR and terahertz radar remain open research challenges. Overall, the results suggest that the future of autonomous vehicle perception will depend not only on improvements in individual sensors but also on robust sensor fusion architectures, safety-certified AI models and energy-efficient sensor processing platforms. These findings provide guidance for researchers and engineers developing next-generation sensing systems for autonomous driving.

## 1. Introduction

Autonomous vehicles represent one of the most rapidly evolving technological fields in modern transportation. Advances in sensing, artificial intelligence and embedded computing have enabled vehicles to perceive their environment, interpret complex traffic situations and assist or partially replace human drivers in decision-making processes. These developments have the potential to significantly improve road safety, reduce accidents caused by human error and optimise traffic efficiency. However, the safe operation of autonomous vehicles fundamentally depends on the reliability and robustness of the sensing systems that provide information about the surrounding environment [1]. The level of automation in road vehicles is commonly classified according to the SAE J3016 standard, which defines six levels of driving automation ranging from Level 0 (no automation) to Level 5 (full automation) [2]. At lower levels of automation, the human driver remains responsible for most driving tasks while the vehicle provides limited assistance, such as adaptive cruise control or lane keeping. At higher levels, the automated driving system assumes increasing responsibility for environmental perception, decision-making and vehicle control. In Level 4 and Level 5 systems, the vehicle must independently detect lane markings, traffic signs, pedestrians, other vehicles and static obstacles, while simultaneously estimating its own position and motion. Achieving this capability requires sophisticated sensing architectures capable of operating reliably under a wide range of environmental conditions (Table 1).

In practice, no single sensor technology is capable of providing robust perception in all driving situations. Camera systems offer rich visual information that support object recognition and scene interpretation but are sensitive to lighting conditions and weather disturbances. LiDAR sensors provide accurate three-dimensional geometric information about the environment, but their performance can degrade in heavy rain, fog or snow. Radar sensors, on the other hand, operate reliably in adverse weather and directly measure object velocity, although their spatial resolution is generally lower than that of optical sensors. For this reason, modern autonomous vehicles rely on multi-sensor architectures that combine complementary sensing modalities to achieve reliable perception. Over the past decade, sensor technology has developed rapidly [3,4]. Camera systems now offer higher resolution, improved dynamic range and enhanced night vision capabilities. LiDAR sensors have evolved from large mechanical scanning devices toward compact solid-state and integrated photonic systems. Radar technology has also advanced with the emergence of imaging radars and experimental terahertz radar concepts. At the same time, artificial intelligence methods—particularly deep neural networks and transformer-based architectures—have dramatically improved the ability of vehicles to interpret sensor data in real time. Despite these advances, autonomous driving systems still face significant challenges related to environmental variability, sensor limitations, computational requirements and functional safety constraints [5].

**Figure 1:**
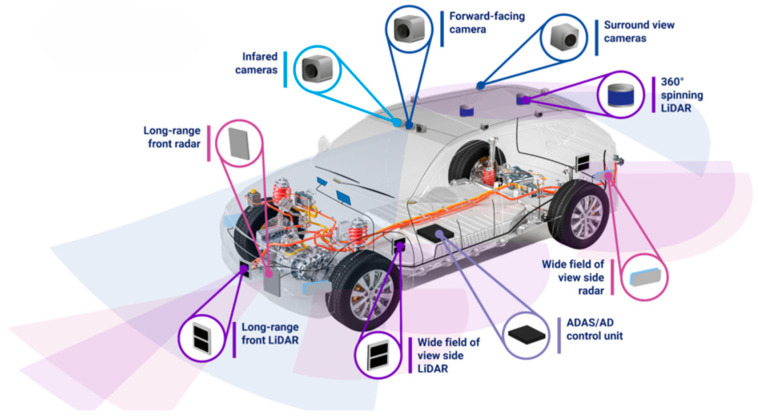
Overview of the main sensor types used in autonomous vehicles. The illustration shows the typical placement of cameras, LiDAR, radar and ultrasonic sensors around the vehicle body. Cameras are usually mounted behind the windshield and on the vehicle perimeter to capture visual information, LiDAR sensors provide three-dimensional point clouds of the environment, radar modules detect objects and measure relative velocity, while ultrasonic sensors are used for short-range obstacle detection during parking and low-speed manoeuvres. Source: [6].

A large number of studies have investigated individual sensing technologies used in autonomous vehicles. Previous review papers, including those by Yeong et al. and Qian et al., [3] provide valuable overviews of specific sensor modalities or perception algorithms. However, many existing surveys primarily focus on describing individual sensors rather than analysing the system-level trade-offs that arise when these sensors are integrated into complete autonomous vehicle architectures. In particular, relatively little attention has been given to the interaction between sensing performance, environmental robustness, computational requirements, cost constraints and functional safety considerations. The aim of this study is to provide a comprehensive and critical review of sensing technologies used in autonomous vehicles, with particular emphasis on system-level integration and practical deployment challenges. In contrast to previous surveys that mainly summarise sensor specifications, this paper analyses the strengths and limitations of different sensing technologies in real-world operating conditions and evaluates their implications for autonomous vehicle design. Special attention is given to emerging sensing technologies such as neuromorphic event cameras, frequency-modulated continuous-wave (FMCW) LiDAR systems and terahertz radar, which may significantly influence the next generation of autonomous perception systems (Figure 1).

The main contributions of this paper can be summarised as follows:

- A comprehensive taxonomy of sensing technologies used in autonomous vehicles, including cameras, LiDAR, radar, ultrasonic sensors, localisation systems and V2X communication.
- A comparative analysis of sensor performance with respect to environmental robustness, detection range, resolution and operational limitations.
- A discussion of system-level trade-offs between sensing performance, computational requirements and energy consumption.
- An examination of functional safety considerations and reliability challenges associated with autonomous sensing systems.
- Identification of emerging sensing technologies and open research questions relevant to the future development of autonomous driving systems.

The remainder of the paper is organised as follows. Section 2 presents the methodology used for the systematic literature review and summarises related research in the field. The following sections analyse the main sensing technologies used in autonomous vehicles, including visual sensors, LiDAR systems, radar technologies and ultrasonic sensors. Subsequent sections examine localisation technologies based on IMU and GNSS integration, as well as sensor fusion architectures used to combine heterogeneous sensor data. Finally, the concluding sections summarise the main findings of the review and outline future research directions for autonomous vehicle sensing systems [7].

## 2. Review Methodology

Before presenting the methodology of the literature review, it is useful to briefly position the present study relative to previous survey papers in the field. Several recent review articles have examined sensing technologies used in autonomous vehicles. For example, Yeong et al. provided an overview of perception systems for automated driving with a focus on sensor architectures and perception algorithms, while Qian et al. analysed the characteristics and limitations of LiDAR, radar and camera-based sensing technologies in autonomous vehicles [3]. Although these studies offer valuable insights into individual sensing modalities, many existing surveys primarily describe the technical properties of sensors without systematically analysing their integration challenges, environmental robustness and system-level trade-offs in practical autonomous vehicle architectures. In addition, emerging sensing technologies such as event cameras, FMCW LiDAR and terahertz radar are only partially covered in earlier surveys. The present review therefore aims to complement existing work by providing a broader comparative analysis of sensing technologies used in autonomous vehicles, with particular emphasis on system-level design considerations, sensor fusion implications and emerging sensing paradigms that may influence the next generation of autonomous perception systems.

**Figure 2:**
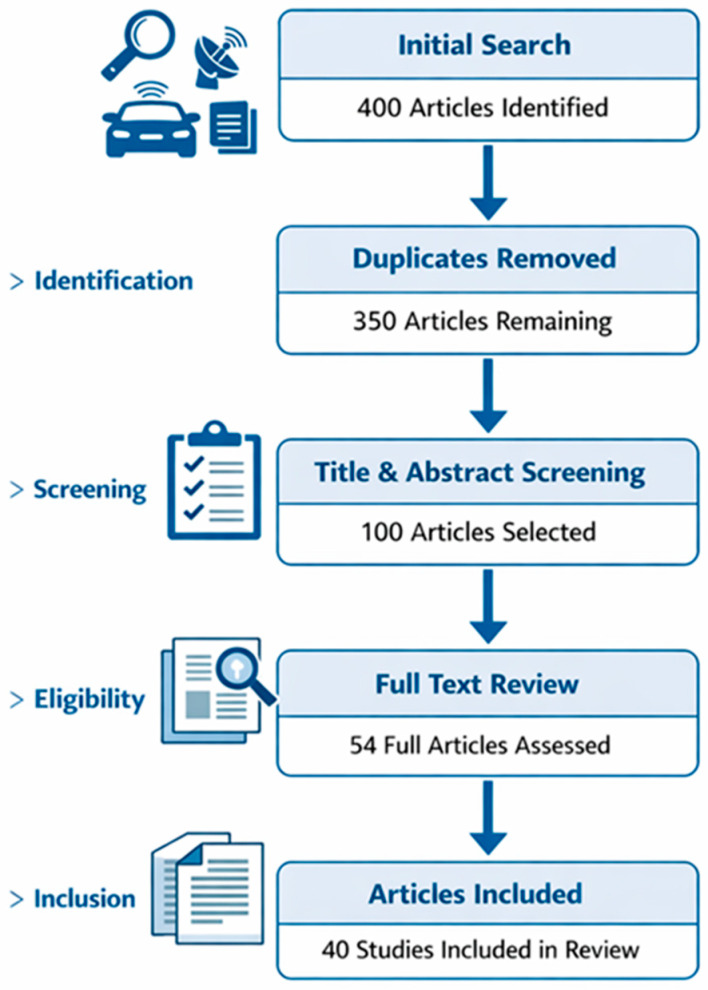
Flowchart of the literature search and evaluation process. The initial database search produced several hundred publications. After removing duplicates and screening titles and abstracts, 40 primary studies were selected for detailed evaluation and comparative analysis. Additional references were included in the manuscript to provide background context and discussion of emerging technologies relevant to autonomous vehicle sensing systems. Source: own edited.

We used a systematic search strategy to prepare the literature review. As a first step, we compiled a list of keywords covering the entire spectrum of sensors used in autonomous vehicles: “autonomous vehicle sensors”, “camera LiDAR radar ultrasound”, “sensor fusion”, “sensor errors”, “FMCW LiDAR”, “terahertz radar”, “V2X communication”. We searched for publications between 2020 and 2026 using these terms in the Google Scholar, IEEE Xplore, Scopus, Web of Science and MDPI Sensors databases. Each search yielded hundreds of results, so it was necessary to remove duplicates and filter the results according to relevance. We adjusted the search process based on the source indicated in He et al. [8], which presents the established practice of selecting multi-sensor fusion publications [9]. It is important to distinguish between the primary studies included in the systematic review and the additional references cited in the manuscript. The systematic comparative analysis presented in this paper is based on 40 primary studies selected through the described search and screening process. Additional references were included to provide background information on sensor technologies, algorithmic developments and emerging research trends. Consequently, the total number of references in the bibliography exceeds the number of studies included in the systematic review (Figure 2).

Filtering and selection criteria: We removed duplicates from the preliminary results, as well as articles on non-autonomous vehicles (e.g., general robotics or industrial applications) if they did not contain transferable methodological lessons. We examined the relevance of the articles based on their titles and abstracts: we only considered papers that specifically dealt with sensor performance, comparison or fusion. We excluded publications that contained purely conceptual proposals or simulations without experimental validation. During the acceptance process, we took into account technical novelty, measured range, resolution, environmental robustness, and functionality. Data extraction and classification: We created a structured data sheet from the selected articles, recording the sensor type, operating principle, specifications (range, resolution, viewing angle), testing environment, and main results. During the investigation, we paid particular attention to the effects of weather conditions (rain, fog, snow), lighting (daytime, dusk, night-time) and dynamic environments (city traffic, motorways), as these conditions often cause a decline in sensor performance. Based on the data sheets, we formed thematic groups: cameras (conventional, HDR, multispectral, event cameras, thermal cameras), LiDAR systems (scanning, solid-state, FMCW), radars (millimetre wave, 4D imaging, terahertz), ultrasonic sensors, localisation sensors (GNSS, IMU) and V2X communication devices. When developing the thematic grouping and data sheet structure, we took into account the testing methodology of open-access automotive benchmarks (KITTI, nuScenes) and reviews organised by application area to ensure comprehensive coverage of relevant testing criteria [10,11].

### Evaluation Method

Each article was independently evaluated by two researchers in order to reduce subjective bias during the selection and assessment process. The evaluation was performed according to five predefined criteria: (1) technical reliability of the experimental methodology, (2) degree of innovation, (3) relevance to autonomous vehicle sensing systems, (4) reproducibility of the reported results, and (5) overall contribution to the research field. To ensure consistency between the evaluators, a pilot evaluation was first conducted on a subset of ten randomly selected articles. The agreement between the two reviewers was measured using Cohen’s kappa coefficient, which resulted in κ = 0.8185, indicating a high level of inter-rater reliability. After this calibration phase, the full set of selected publications was evaluated independently [12]. The overall agreement rate between the two evaluators during the full review process was approximately 88%. In cases where the two reviewers assigned different quality ratings to a paper, the disagreement was resolved through joint discussion and re-examination of the methodology and reported results [13]. If consensus could not be reached immediately, the reviewers revisited the predefined evaluation criteria and reassessed the article until agreement was achieved [14,15].

This two-stage evaluation procedure ensured that the literature selection and quality assessment process remained systematic, transparent and reproducible. In compiling the comparative tables, we took into account recent studies on the weather dependence of LiDAR reflection characteristics [16,17], sensor calibration challenges [18,19], adaptive radar signal and parameter processing [20,21], multi-sensor 3D object detection algorithms [22], and terahertz radar research [23]. These publications highlight the trade-offs between parameters and contributed to the development of the tables.

## 3. Visual Sensors

### 3.1. Traditional Cameras and Image Processing

Cameras provide the system with colour and textural information that is essential for recognising road surfaces, lane markings, traffic signs, pedestrians and other objects. According to a summary by DPV Transportation, cameras provide rich image data that is used by deep neural networks (e.g., convolutional neural networks, YOLO, Mask R-CNN) for object detection and classification tasks. However, traditional RGB cameras are sensitive to lighting conditions (strong sunlight, night-time conditions) and weather phenomena that obstruct vision (fog, rain) [24]. In addition, in monocular form, they lack depth information, which must be supplemented by optical flow or artificial intelligence-based depth estimation. Developments in recent years have significantly improved camera performance. High dynamic range (HDR) cameras allow dark and light areas to appear in detail in the same image; night vision infrared systems detect heat radiation beyond the visible spectrum; and spectrometric cameras record data in multiple wavelength ranges. Hybrid RGB-IR cameras operate on two spectra simultaneously, providing useful information even at night [25]. Deep learning-based software solutions enable image quality improvement in low light conditions through noise reduction and super-resolution reconstructions.

#### 3.1.1. Depth Sensing with Cameras

Monocular and stereo cameras estimate depth using different methods. Stereo cameras have two or more lenses; epipolar geometry can be used to calculate a three-dimensional point cloud from the parallax difference between the two images. Structured light systems project a pattern onto the scene and calculate distance from the distortions. Monocular depth estimation uses neural networks (e.g., MiDaS, DPT) to learn the spatial distribution of depth. These methods do not yet achieve the accuracy of LiDAR, but they are an attractive alternative due to lower hardware costs.

#### 3.1.2. Data Volume and Processing

High-resolution cameras generate huge amounts of data, which places significant computational and memory demands on on-board systems. A 12-megapixel camera at 30 frames per second outputs approximately 10–15 Gb/s of raw data. Compressing, decoding and processing this data requires dedicated graphics processing units (GPUs), tensor processing units (TPUs) or neural accelerators. To reduce energy consumption, modern systems use selective attention and frame skipping techniques.

### 3.2. Neuromorphic Event Cameras

Neuromorphic event cameras represent a revolutionary new approach to image sensing. Individual pixels send data independently and asynchronously only when their brightness exceeds a predetermined threshold. In practice, each pixel records the time derivative of the logarithmic intensity change and then generates an “event” that contains the pixel location, timestamp, and polarity (positive or negative change). This operation has several advantages. Microsecond delay: the event camera achieves a temporal resolution of 1–10 µs, which is several orders of magnitude faster than the 10–100 ms refresh time of conventional cameras [26]. This allows fast movements to be tracked and fast control loops to be implemented. Extremely high dynamic range: logarithmic encoding allows for a dynamic range of up to 120–140 dB, compared to around 60 dB for conventional cameras [27]. This allows the sensor to detect dark and bright areas simultaneously, avoiding burnout and underexposure. No motion blur: since the sensor does not capture entire frames, but only changes, there is no blurring caused by movement [28]. Low energy consumption: data transfer is proportional to the number of changes occurring in the scene; in static scenes, the sensor generates minimal data, resulting in energy consumption of between 10 and 100 mW [29]. The disadvantage of event cameras is that they do not measure absolute intensity, so special algorithms are needed to reconstruct the information. Hybrid systems (e.g., DAVIS) combine event output and intensity imaging in a single sensor, enabling the combined use of classical and neuromorphic sensing. According to the 2026 Emergent Mind report, the latest products, such as the Prophesee EVK4 (1280 × 720 px), have a dynamic range of 140 dB, and processing of a microsecond event timestamp’s event data differs from traditional image processing: voxel grid representations, time surfaces, and learned event tensors are common [30,31]. These representations allow convolutional or transformer-based neural networks to learn directly from the event stream.

Research into neuromorphic cameras is constantly expanding: special SLAM algorithms, energy-efficient chip architectures and hybrid sensing models are being developed for real-time object detection and localisation. Recent studies investigate the combined use of event cameras and traditional cameras for high-speed navigation [32,33,34] and propose energy-efficient image processing units for high-resolution cameras [35]. Event-based methods have also appeared in camera–LiDAR fusion, which utilise sparsity to reduce latency and increase accuracy [36].

### 3.3. Thermal and Infrared Cameras

Thermal cameras detect heat radiation in the infrared (IR) range, typically at wavelengths of 8–14 µm. Since the emitted infrared radiation is proportional to the temperature of objects, the thermal image is independent of visible light conditions and can therefore be used at night or in poor visibility conditions. According to Teledyne FLIR’s announcement of 2026, the Tura LWIR camera is the first thermal camera module to meet the ASIL-B level of the ISO 26262 functional safety standard [36]. The Tura has a passive sensor with a resolution of 640 × 512 pixels and can detect heat-emitting objects (pedestrians, animals, engine blocks) in complete darkness, fog or bright sunlight [37]. The system has an integrated heating cover that prevents lens fogging and ice formation and uses a shutterless design for faster response times [38]. Several studies have analysed the advantages of dual-channel (RGB + IR) sensing in detail: coordinated processing of thermal and visible images can significantly improve pedestrian detection and distance estimation accuracy at night and in poor visibility conditions [39,40]. These studies present IR-RGB fusion network architectures and the challenges of multimodal learning in autonomous vehicles.

The disadvantages of infrared cameras are their higher price and limited resolution. Compared to the visible range, the images are poorer in texture, requiring additional work from recognition algorithms. The latest research aims to coordinate the processing of dual-channel (RGB + IR) camera systems, where deep neural networks use the output of both sensors together to improve pedestrian recognition and distance estimation.

The table below summarises the most important characteristics of visual sensors, highlighting their range, dynamic range, advantages and disadvantages.

Table 2 highlights that while RGB cameras provide rich data, specialised cameras (HDR, event, thermal) play a key role in extreme conditions. In practice, these sensors work in combination to ensure reliability.

The values presented in Table 2 represent typical ranges reported in the recent literature and industrial specifications. Actual detection performance depends strongly on environmental conditions, sensor resolution and object characteristics.

### 3.4. Evaluation of Visual Sensors

To avoid redundancy, the detailed technical characteristics of RGB, event and thermal cameras presented in the previous subsections are not repeated here. Instead, this section focuses on system-level evaluation and integration challenges of visual sensors in autonomous vehicle perception systems. Visual sensors are the primary “eyes” of autonomous vehicles. This category includes traditional RGB cameras, high dynamic range (HDR) and multispectral cameras, neuromorphic event cameras, and thermal and infrared cameras. Although all types serve to provide a visual representation of the environment, they are based on different physical principles and technological parameters. The aim of the evaluation is to explore the extent to which these sensors contribute to autonomous navigation, their strengths and weaknesses, and the research and development directions that are needed in the future. HDR and multispectral cameras aim to mitigate lighting problems. HDR systems combine multiple exposure levels into a single image, displaying both dark and light areas simultaneously and minimising burnout. Multispectral cameras also detect wavelengths that are invisible to the human eye (near-infrared, short-wave infrared), which improves contrast in fog, rain or at night. The disadvantage of these technologies is that the high dynamic range requires multiple images to be captured or a special sensor architecture, which increases data transfer and processing requirements. Multispectral cameras often offer lower spatial resolution because the sensor pixels must be shared between multiple colour bands. In addition, system calibration is complex: the coordination of different exposures and wavelengths is time- and temperature-dependent. One direction for future research is the development of adaptive exposure algorithms that optimise sensor dynamics in real time depending on the environment [41]. The task for the future is to create large event databases and develop event-processing neural networks that can take full advantage of asynchronous data streams. Due to the large sensor elements and special detector materials, the price is higher, and the resolution of thermal cameras often does not reach that of RGB cameras. Future developments aim to develop high-resolution IR sensors covering multiple spectra, as well as artificial intelligence-based algorithms for the combined processing of IR and RGB images [42].

It is important to note that data from visual sensors is not processed in isolation. Deep learning models often use multimodal input, where the RGB image, depth map, event stream and IR image are fed into the network simultaneously [43]. This not only exploits the complementarity of the sensors but also increases robustness: if the data quality of one sensor deteriorates (e.g., in case of rain), the other sensor can compensate. Transfer learning and domain adaptation techniques can help models perform well despite changing conditions (day–night, different geographical regions, weather). At the same time, data sets are often biassed: dense fog, snowfall, or long tunnel sections are rarely represented in the training of autonomous systems. Therefore, real-time data collection in diverse environments and the simulation of sensor errors are critical. The integration of visual sensors into the vehicle’s hardware architecture can cause significant energy consumption and heat load. A modern camera and its associated signal processing SoC (System on Chip) can require up to 5–10 W of power. With dozens of cameras, cooling and power supply are not trivial tasks. In addition, the placement of cameras is limited from an aerodynamic point of view: they must be mounted high to cover the field of view, but this makes them more exposed to contamination and mechanical damage. Manufacturers are therefore developing concealed modules that are located behind parts of the bodywork, behind a transparent cover, while ensuring that they can be kept clean. Another area of research is the development of privacy-conscious camera architectures. Since cameras also record human faces and number plates, users may be concerned about data privacy abuses. Built-in encryption, edge computing and context-based anonymisation are solutions that can alleviate these concerns. In addition, systems must be able to detect attacks such as blinding laser light or stickers placed over sensors. Self-checking diagnostics for sensors, redundant cameras, and continuous calibration of sensors can help maintain reliability.

An evaluation of visual sensors shows that, although these sensors are essential for understanding the environment, they are not sufficient on their own. Colour and high-resolution images enable complex recognition tasks, but changing light conditions and weather pose significant challenges. Event cameras and thermal sensors partially solve vision problems, but their integration is still in its early stages. On the software side, it is essential to collect large, representative data sets, develop domain adaptation algorithms, and ensure model safety. In future systems, visual sensors will operate as part of a sensor fusion network, where information from different spectra is merged to provide reliable, timely and energy-efficient detection.

**Figure 3:**
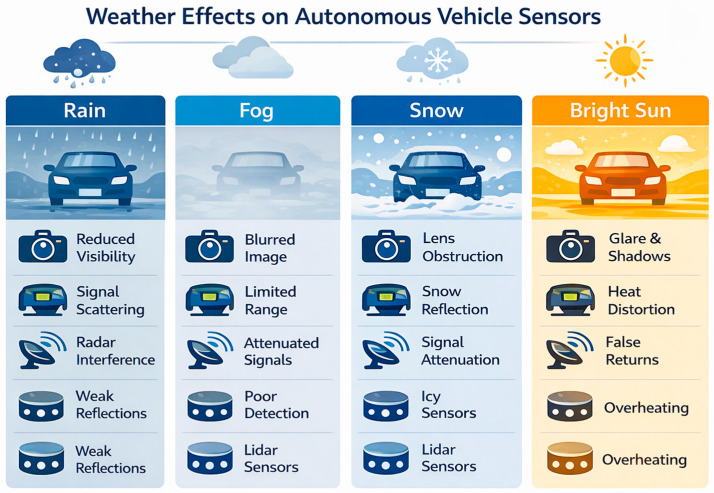
Influence of environmental conditions on camera-based perception systems. Weather phenomena such as rain, fog, snow and direct sunlight can significantly degrade image quality by reducing contrast, causing glare or partially blocking the camera lens. These effects may reduce object detection accuracy and require compensation through sensor fusion or image enhancement algorithms. Source: own edited.

To avoid unnecessary repetition, the detailed technical characteristics and limitations of visual sensors discussed in the previous section are not repeated here. Instead, this section focuses on integration challenges and system-level considerations related to the use of visual sensing in autonomous vehicles. In practical autonomous driving systems, cameras rarely operate as standalone sensors. Their outputs are typically integrated with LiDAR, radar and localisation sensors through sensor fusion frameworks. This integration improves reliability in situations where camera performance may degrade due to adverse lighting conditions or environmental contamination. Therefore, the main engineering challenge is not only the development of better cameras but also the effective integration of visual data into multi-sensor perception architectures (Figure 3).

Finally, it is worth mentioning future development directions and criticisms. Camera resolution has already reached 8–12 megapixels, which is sufficient for most automotive applications. Further growth is likely to be in the direction of spectral diversity: multi-spectral cameras (SWIR, NIR, polarimetric) may be the key to the future, especially in poor visibility conditions. Event camera-based detection is currently expensive, and software processing is complex, but the potential is enormous. The price of thermal cameras is expected to fall as production volumes increase and integrated systems (RGB + IR) become more widespread. The biggest criticism of camera-based systems is that they are sensitive to a single sensor failure and have difficulty meeting automotive safety integrity level (ASIL) requirements. Redundancy, sensor fusion and software control serve to mitigate this. The development of visual sensors therefore seeks a balance between cost, performance, reliability and power consumption. This critical examination also highlights that the greatest challenge for visual sensors is the diversity of the autonomous driving environment. Cameras detect objects at different distances, with varying textures and colour ranges; at the same time, motorways, city roads, country roads, gravel paths, tunnels and viaducts create different lighting and shading conditions. This is compounded by cultural differences: road signs in China, America, Europe and Africa are different. Most of the current data sets come from Western infrastructure, so deep learning models often fail in a global environment. The technological and social task for the future is to map this diversity and adapt sensor hardware and algorithms across the globe. When evaluating visual sensors, it is therefore essential to take into account geographical and cultural diversity, which is key to ethical and reliable autonomous vehicle development. Recent research has highlighted that errors in camera-based systems often depend on how well the training data represents real-world conditions. Analysis of real-world accidents has shown that algorithms that are overly optimised for good weather and clearly visible signs tend to overlook unusual objects or partially obscured pedestrians. Several studies have shown that perception systems trained on limited data sets may fail to recognise unusual objects or rare traffic scenarios, particularly when these situations are underrepresented in training data. This highlights the importance of collecting diverse data sets that include uncommon road users, atypical vehicles and region-specific traffic situations. Regular and open data sharing and relevance-based training are important steps in increasing the reliability of cameras.

**Figure 4:**
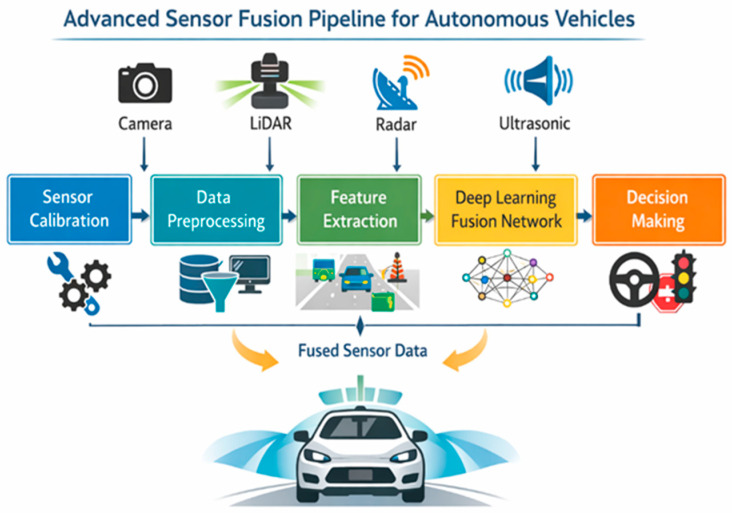
Examples of environmental disturbances affecting visual sensors in autonomous vehicles. The figure illustrates how precipitation, lens contamination or strong backlighting can reduce the reliability of camera-based object detection systems, highlighting the importance of combining cameras with complementary sensors such as LiDAR or radar. Source: own edited.

Lifespan and maintenance are also critical to the long-term operational reliability of camera systems. Lenses are subject to constant stress: UV radiation, chemical contamination, minor scratches and thermal fluctuations gradually degrade optical performance. Modular camera design is becoming increasingly common in the industry, with camera capsules that can be quickly replaced and lens protection glass with a hydrophobic coating. In the future, the lifespan of cameras may also be extended by self-monitoring diagnostics that track changes in resolution, exposure, and brightness and provide timely maintenance alerts. These critical considerations suggest that camera development should focus not only on increasing megapixels but also on software robustness, hardware durability and data set diversification (Figure 4). Visual sensors can only fulfil their key role in autonomous vehicles if artificial intelligence algorithms are trained on data sets that reflect global traffic culture and extreme conditions.

### 3.5. LiDAR Sensors

LiDAR is short for “Light Detection and Ranging”. The system emits a laser beam that is reflected by the surface of objects, and the distance can be calculated based on the return time and the speed of light. The scanning is performed by rotating mirrors, MEMS micro-mirrors or semiconductor modules. The LiDAR point cloud contains millions of points that form a three-dimensional map. The range and resolution of the devices vary depending on the type. Traditional mechanical LiDAR systems use a rotating mirror or laser beam to scan the environment. Their disadvantages are reliability issues due to moving parts and high cost [3]. Solid-state LiDAR systems use MEMS mirrors, silicon photonic modules or optical phase-controlled gratings, which reduce the number of moving parts. Flash LiDAR emits a large, single light pulse, illuminating the entire field of view at once but offering lower resolution. FMCW (Frequency Modulated Continuous Wave) LiDAR emits a continuously varying frequency signal and calculates both distance and speed from the frequency shift of the returning signal.

Advantages and limitations:

According to an article in DPV Transportation, LiDAR devices provide 360° coverage and centimetre-level accuracy, making them ideal for real-time mapping and SLAM tasks [24]. The point cloud is independent of visible light, so it can also be used at night. LiDAR can measure not only distance but also reflected signal strength, which helps with object classification.

Disadvantages:

High cost and complexity: traditional rotating systems are expensive, and mechanical components are prone to failure. Therefore, developments are moving towards solid-state systems.

Sensitivity to weather: rain, fog and snow scatter and absorb the laser beam, reducing the range and strength of the returned signal [3]. The degree of signal distortion depends on droplet size, precipitation intensity and wavelength, as shown by the attenuation factors for wavelengths of 905 nm and 1550 nm [4]. Lack of colour information: LiDAR only measures distance and intensity; it must be combined with cameras to supplement colour information.

#### 3.5.1. FMCW LiDAR the Next Generation

Frequency-modulated continuous wave (FMCW) LiDAR offers a significant advance over traditional time-of-flight (ToF) systems. Frequency-modulated continuous wave (FMCW) LiDAR offers improved distance resolution compared to traditional time-of-flight systems. Modern FMCW implementations developed by companies such as Bridger Photonics demonstrate the ability to measure both distance and radial velocity through Doppler frequency analysis, enabling true four-dimensional point cloud generation.

Recent industry demonstrations have highlighted the growing interest in integrated FMCW LiDAR architectures. For example, several prototype systems presented at technology exhibitions integrate lasers, modulators and detectors on silicon photonic chips, enabling compact and potentially lower-cost LiDAR sensors.

#### 3.5.2. Terahertz Radar the “Teradar” Concept

Terahertz radar systems represent an experimental extension of conventional millimetre-wave radar technology. While current automotive radars typically operate in the 24–81 GHz range, terahertz radar concepts aim to use frequencies between approximately 0.3 and 3 THz. Increasing the carrier frequency significantly reduces the wavelength, which theoretically improves angular resolution. According to reports discussed in the automotive technology literature, prototype terahertz radar systems may achieve approximately a 13× improvement in angular resolution compared with conventional 77 GHz radar systems under comparable antenna aperture conditions [10]. Other experimental studies suggest that the effective spatial resolution of certain terahertz radar architectures could approach or exceed the resolution of current automotive radar systems by up to an order of magnitude, although these estimates are typically derived from simulation results or early laboratory prototypes rather than fully validated automotive deployments [11]. It is important to note that these reported improvements depend strongly on several factors, including antenna size, signal bandwidth, environmental conditions and signal processing methods. Consequently, the performance advantages of terahertz radar should currently be interpreted as theoretical or prototype-level results rather than mature automotive technology. Further experimental validation and large-scale testing are required before such systems can be deployed in commercial vehicles.

##### Range and Resolution Comparison Table

The table below compares different distance measurement sensors, taking into account detection range, point resolution, speed measurement and weather sensitivity. The values are based on typical data given in the literature.

Beyond the basic technological categories discussed above, it is also important to evaluate LiDAR systems from a system-level perspective. The following discussion therefore focuses on practical engineering challenges such as weather robustness, computational requirements and safety considerations. Weather resistance is crucial for LiDAR systems. Rain, fog and snow attenuate and scatter the laser beam due to Mie scattering: the 905 nm and 1550 nm wavelengths are affected to varying degrees [4]. Research shows that the 1550 nm wavelength is less sensitive to fog, but the detectors are more expensive; in contrast, the 905 nm system is cheaper but suffers from a drastic reduction in range [12]. Manufacturers are trying to combine multiple wavelengths or increase the signal-to-noise ratio with adaptive amplification. Furthermore, large raindrops can cause false reflections; the system must distinguish between noise from precipitation and real objects. These problems force developers to combine LiDAR with camera and radar data and to use real-time rain and fog compensation algorithms in signal processing. Quantitative measurements reported in the literature show that atmospheric attenuation strongly depends on both wavelength and environmental conditions. Table 3 summarises typical attenuation coefficients for 905 nm and 1550 nm LiDAR systems under different weather scenarios.

The data indicate that longer wavelengths around 1550 nm generally exhibit lower attenuation in fog and precipitation compared to 905 nm systems. However, the improved weather robustness of 1550 nm LiDAR comes at the cost of more expensive InGaAs detectors and higher system complexity (Table 4).

In addition to attenuation coefficients, several studies report measurable reductions in LiDAR detection range under increasing precipitation intensity. For example, experimental measurements indicate that the effective detection range of a long-range automotive LiDAR sensor can decrease from approximately 2 km in clear atmospheric conditions to around 1.2 km during moderate rainfall of approximately 2 mm/h. Under heavier precipitation or dense fog conditions, the effective detection distance may decrease even further due to increased scattering and absorption of laser pulses by water droplets and aerosols. To illustrate this effect more clearly, Table 4 presents a conceptual relationship between precipitation intensity and LiDAR detection range degradation based on representative values reported in the literature.

LiDAR data processing is also a significant challenge. A 64-line system can generate 2 to 4 million points per second, and higher-end systems can generate even more. Segmenting, clustering, and classifying the data requires significant computational power, especially when deep learning models are used to process point clouds. Three-dimensional convolutional neural networks, voxel-based, BEV (bird’s eye view) and point cloud grid representations all involve different trade-offs between accuracy and computational requirements. For real-time processing, the LiDAR data stream is often downsampled or concentrated on areas of interest. Future integration of the devices with on-board GPUs and AI accelerators is expected, but even so, high energy consumption and heat generation will still be an issue. Safety and regulatory issues related to LiDAR systems cannot be ignored either. Laser power must be limited to protect human eyes and skin; accordingly, LiDARs used in the automotive industry employ Class I lasers. Another problem is mutual interference: if two vehicles’ LiDAR systems operate at the same wavelength and use the same modulation, the sensors can interfere with each other. LiDAR manufacturers therefore introduce unique coding, time division or random frequency hopping. In addition, cybersecurity is important because the LiDAR data stream can be manipulated; for example, false reflectors (spoofing) can be created. The system must be able to detect and counter such attacks (Table 5).

Finally, further development of LiDAR technology is moving in several directions. Researchers are investigating metamaterial-based antennas and quantum detectors to increase detection efficiency. Photon-counting detectors can detect even weak reflections, which increases the range and reduces blind spots. On the processing side, hybrid LiDAR–camera calibration and neural ray tracing are opening up new horizons: artificial intelligence models learn the relationship between LiDAR intensity and camera colour information, generating coloured point clouds. Software and hardware developments are taking place in parallel, and both are essential for LiDAR to remain one of the key sensors in autonomous vehicles in the future.

### 3.6. Radar Systems

Radar sensors complement LiDAR and camera systems by providing reliable detection capabilities under adverse environmental conditions. Unlike optical sensors, radar operates using radio waves, which are significantly less affected by rain, fog or dust. In addition, radar sensors directly measure object velocity through the Doppler effect, which makes them particularly useful for collision prediction and high-speed driving scenarios.

Wavelength and optical power are important considerations for LiDAR systems. Most commercial LiDARs use 905 nm diodes because they are inexpensive and the detector (Si APD) is sensitive to them. However, at 905 nm, the output power must be limited to protect the eye, which reduces the range in bad weather. Fibre lasers of 1550 nm allow higher power to be emitted without damaging the eye, thus increasing range and signal-to-noise ratio [4]. However, 1550 nm systems are more expensive, and the InGaAs detectors on the receiver side are costly. The choice of wavelength reflects a compromise between cost, safety and performance. Multi-spectral LiDAR systems, which operate at multiple wavelengths, can provide additional information about the material and texture of the target, but processing is more complex. The processing and interpretation of LiDAR point clouds is also a critical area. Flat surfaces (walls, roadways), three-dimensional boundaries and moving objects must be extracted from the raw point cloud. In addition to classic algorithms (RANSAC, region growing, Euclidean clustering), deep learning-based models (PointNet, PointPillars, VoxelNet, 3D Swin Transformer) are playing an increasingly important role. These models are capable of learning the structure of point clouds, but they require high computing power and rely on FPGAs or special AI accelerators for real-time execution. Multi-modal fusion architectures (e.g., BEVFusion, FusionTransformer) aimed at processing LiDAR and camera data together have brought significant accuracy improvements, but sensor time synchronisation and calibration are complex [5]. It is also important to mention the issue of environmental effects and interference. The environmental limitations of LiDAR systems have already been discussed earlier in this section. In practice, these limitations are mitigated through sensor fusion approaches that combine LiDAR with radar and camera systems, thereby increasing robustness in challenging weather conditions.

#### 3.6.1. Millimetre-Wave Radars

Radars (Radio Detection and Ranging) use the reflection of radio waves to determine distance and speed. In the automotive industry, they typically operate in the 24 GHz (medium range) and 77–81 GHz (long range) bands. According to a review by DPV Transportation, radars provide long-range detection and speed information while being less affected by rain, fog or dust [24]. Modern 77 GHz radar systems feature phased array antennas consisting of multiple antennas, which enable spatial narrowing and digital beamforming, thereby improving angular resolution.

#### 3.6.2. Short, Medium and Long-Range Radars

The automotive industry uses different radars depending on the task:

Short-range radar (SRR): Operates in the 24 GHz band, in the 0–30 m range, with low angular resolution. Its main tasks are blind spot monitoring, parking assistance and cross-traffic monitoring.

Medium-range radar (MRR): Operates in the 76–77 GHz band, with a range of 30–80 m. Typically used to support lane keeping and adaptive cruise control (ACC) systems.

Long-range radar (LRR): Operates in the 77–81 GHz band and detects up to 250 m. It features higher angular resolution, multiple antenna arrays, and more complex signal processing, which is suitable for collision prediction and high-speed driving assistance.

### 3.7. Limitations and Developments

Although radars perform well in adverse weather conditions, their spatial resolution is lower than that of LiDAR, resulting in a sparser point cloud that is more difficult to interpret [6]. Vehicle manufacturers are improving this with larger antenna arrays, complex modulations (FMCW, MIMO) and machine learning-based super-resolution algorithms. According to a report by MotorTrend, Neural Propulsion Systems (Atomathic) is developing an AI algorithm that generates multiple hyper-step hypotheses from raw radar reflections and then selects the most likely object based on physical models [10]. The goal is to display radar points in a denser and more informative way, closer to LiDAR point clouds. The future of autonomous vehicle radars is linked to frequency increases and the spread of 4D imaging radars. Radars operating at terahertz frequencies, such as Teradar, are characterised by ultra-high bandwidth and high angular resolution, resulting in a lidar-like point cloud [10]. These systems perform electronic beam scanning without moving parts, making them more durable and easier to integrate. According to an article in IEEE Spectrum, terahertz radars offer up to 20× resolution and a “superset” of radar and LiDAR functions, while potentially being less expensive than traditional LiDAR systems [11]. Another trend is 4D imaging radars, which determine three-dimensional position and velocity in addition to time. These systems use large antenna arrays, MIMO (Multiple-Input Multiple-Output) configurations, and high-bandwidth FMCW modulation. This makes the radar point cloud denser, which helps machine learning algorithms to recognise objects more accurately. Radars in autonomous vehicles play a unique role in environmental perception, as they measure distance and speed in real time based on the reflection of electromagnetic waves. A key advantage of radars is that they operate reliably even in bad weather, as millimetre-wave and terahertz frequencies are less sensitive to rain and fog [7,10]. However, radar technology poses a number of challenges: spatial resolution is limited, scattered signals and multipath reflections can result in false targets, and the increasing number of vehicles can cause interference in radar bands.

Different categories of radar have different functions: short-range radar (SRR) can be used for blind spot monitoring and parking assistance; mid-range radar (MRR) can be used for lane keeping and adaptive cruise control functions; while long-range radar (LRR) can cover a range of up to 250 m, enabling early detection of frontal collisions. Radars operating at 77–81 GHz use higher bandwidths and MIMO antenna arrays, which improve angular resolution. Nevertheless, radar point clouds are still sparser than LiDAR point clouds, so machine learning and super-resolution signal processing play a key role in enriching radar information [10]. The new generation of imaging radars and 4D radars represents a significant step forward. These systems have large antenna arrays and wide bandwidths, making the radar point cloud denser and 3D. The radar thus determines not only distance and speed but also horizontal and vertical angles. Neural Propulsion Systems’ Atomathic AI radar processes the raw spectrum using neural networks and selects probable targets based on physical models [10]. These approaches are promising, but the lack of data sets, high computational requirements and real-time operation pose challenges.

Terahertz radars (0.3–3 THz) operate at radically higher frequencies, resulting in a dramatic increase in angular resolution [10]. Current terahertz radar concepts should be interpreted with caution because many of the reported performance gains are based on prototype demonstrations, analytical estimates or early experimental systems rather than mature automotive products. Published reports suggest that higher carrier frequencies may substantially improve angular resolution relative to conventional 77 GHz radar, but the exact gain depends strongly on antenna aperture, signal bandwidth, processing method and measurement conditions. For this reason, terahertz radar should currently be described as a promising experimental direction rather than a commercially validated replacement for automotive LiDAR or millimetre-wave radar. THz radars are currently primarily in the experimental stage: the manufacture of terahertz generators, antennas and detectors is complex, and spectrum regulation is still under development. Their future success depends on mass production, cost reduction and reliability testing. Interference can occur during radar operation if multiple vehicles use the same frequency band. The 76–81 GHz band can become crowded, and intersecting radar pulses can result in false signals. To address this, the literature suggests frequency hopping, time division multiple access (TDMA) and coded modulation strategies, but these require international coordination. For terahertz radars, spectrum allocation is even less regulated, which may hinder industrial deployment.

Radars alone are rarely sufficient for object classification; only limited information can be obtained from the intensity (RCS) and time profile of the reflected signal. Camera-radar and LiDAR–radar fusion allows the speed information and all-weather capability of radar to complement the accurate distance measurement of LiDAR and the colour information of cameras. In the future, sensor fusion systems will likely use software-defined radars that dynamically adapt to frequency and time sources and integrate with other sensors via neural networks. From a critical perspective, it is important to note that the hype surrounding the introduction of radar technology often precedes the actual market reality: currently, few commercial 4D or THz radars are available, and most products are in the development phase. The success of the technology requires comprehensive solutions to regulatory, economic and technical challenges.

#### 3.7.1. Micro-Doppler Radar Analysis

Increasing attention is being paid to micro-Doppler signal processing and classification in radars, which enables the identification of different objects and pedestrian movement patterns. The micro-Doppler spectrum contains the frequency modulation of the reflected signal, from which the arm and leg movements of pedestrians, the gait of animals, or the pedalling of cyclists can be filtered out. These patterns are processed by machine learning models, such as supervised classifiers and neural networks. Research shows that micro-Doppler-based recognition can complement LiDAR and camera-based detection, especially in poor lighting conditions. However, micro-Doppler signals are sensitive to the direction of movement and the placement of the radar system, so in practice, radar signals from multiple perspectives need to be fused. The development of radar technology does not stop at 77 GHz. Radar systems operating in the 79 GHz and 150 GHz bands are under development, offering wider bandwidth and better angular resolution. Even higher are terahertz radars around 300 GHz, which can achieve millimetre resolution. These radar systems use new materials (e.g., graphene-based antennas) and integrated circuits, but mass production and cooling still pose serious challenges. Band regulation and the radio spectrum policies of different countries also influence the introduction of terahertz radars.

#### 3.7.2. In-Cabin Radar Sensing

In-cabin applications of radar have also come to the fore in recent years. In-cabin radar uses millimetre-wave sensors to monitor movement and breathing in the passenger compartment, enabling it to detect a baby left in a child seat or a dog sleeping in the back seat of a car. From 2023, the European NCAP assessment system requires vehicles to warn of children left inside; radars perform this function more accurately and reliably than ultrasonic or camera solutions. In addition, radar can be used for touchless gesture control in the passenger compartment, such as hand movement recognition, which increases operating comfort. The economic aspects of radar systems also deserve attention. The price of millimetre-wave modules has fallen significantly over the past decade, but large antenna arrays and high-bandwidth signal processing chips remain expensive. The commercial price of 4D imaging radars and terahertz systems is expected to remain high until production volumes reach critical mass. For car manufacturers, this means a compromise between radar performance and affordability. Radar system developers are seeking to reduce costs through integrated chip solutions and standardised antenna arrays. Finally, it is important to emphasise the data protection and ethical implications of radars. While cameras capture images and LiDAR captures detailed point clouds, radar signals carry less personal data and are therefore more attractive from a data protection perspective. However, the combination of radar point clouds and micro-Doppler spectra has the potential to identify individual walking styles, which could be considered biometric data. Accordingly, developers must ensure that the processing of radar data complies with data protection laws and ethical guidelines and is transparent to users.

#### 3.7.3. Radar Cross Section (RCS) and Object Reflectivity

An important physical factor in radar information processing is radar cross-section (RCS), which shows how much electromagnetic power an object “appears” to emit to the sensor. The RCS of vehicles, pedestrians and bicycles varies greatly and depends significantly on the material, shape and orientation of the object. For example, a pedestrian wearing dark clothing may produce a low RCS, while the metal surface of a large flatbed truck may produce a high reflection. However, radars have difficulty distinguishing between objects with similar RCS; the reflection patterns of a motorcycle approaching from a certain angle and a pedestrian are often indistinguishable. Deep learning-based radar classification is in its early stages, as radar point cloud annotation is labour-intensive and few public data sets are available. In the future, radar neural networks and self-supervised learning methods may help to recognise hidden patterns. Global regulation of radar bands is a major challenge. The 76–81 GHz frequency band is dedicated to automotive radars in Europe, but parts of it are also used by other industries in the United States. The 57–64 GHz band, for example, is licenced for short-range communications and radars in some countries, so automotive manufacturers are careful to choose the usable spectrum to avoid interference. There are currently no uniform international regulations for terahertz (>300 GHz) bands; the lack of authorisation and licence allocation is hindering the commercial introduction of THz radars. Harmonisation of frequency allocation is essential to ensure that future radars can operate without interference in different regions.

The integration of radar modules into vehicle bodies also poses an engineering challenge. Radar antennas must be positioned so that the cover (radome) does not distort the wavefront; this requires the use of special electromagnetically transparent plastics that are UV- and weather-resistant but cause minimal loss. The modules must operate between −40 °C and +85 °C, resisting vibration, moisture and salt spray. Cooling signal processing chips is becoming increasingly difficult with miniaturisation; future integrated radar chips will combine CMOS and SiGe technologies to integrate the entire system onto a single chip, reducing size and cost but requiring new heat dissipation solutions. From an economic standpoint, radar modules are less expensive than LiDAR systems. A basic 77 GHz radar costs a few dozen US dollars, making it affordable for mass-market vehicles; however, 4D imaging and terahertz radars are currently in a higher price range due to complex antenna arrays and high-bandwidth modulation. Car manufacturers often install 6–10 radar modules in a vehicle (forward-looking, rear-looking, blind spot monitoring), which results in higher overall costs. In the future, software-defined radar (SDR) modular platforms will enable feature updates and adaptive use of frequency bands, reducing hardware investment. Radars remain key sensors for autonomous vehicles. The future of the technology depends on balancing higher resolution, global spectrum management, increased computing requirements and costs. Coordinated efforts by industry players—standardisation, open databases, and ethical guidelines—are necessary for radars to truly fulfil their anticipated role in fully autonomous driving.

In addition to this summary, numerous studies address the details of adaptive radar signal processing and the development of advanced systems. Petrovics et al., [30] proposed adaptive signal processing algorithms for automotive radars, while Dai et al. [42] investigated weather-resistant radar systems in urban environments. Weng and Zhang [43] analysed the problems of multi-sensor time synchronisation, while Nabati et al. and Rohling et al. [44,45] modelled LiDAR–radar fusion in adverse weather conditions. Kim and Lim [46] presented deep learning-based FMCW radar signal processing, while Gallego et al., [47] investigated the use of radar micro-Doppler signals to identify vulnerable road users. These works highlight that the future of radar sensors lies in dynamic bandwidth management, learning signal processing, and multi-sensor integration.

### 3.8. Ultrasonic Sensors

Ultrasonic sensors emit sound waves (at a frequency of 20–40 kHz) and calculate distance from the time difference of the reflected signal. These systems are extremely simple and inexpensive, which is why they are widely used as parking radars and short-range obstacle detectors. According to DPV Transportation, ultrasonic sensors have a range of only 1–5 m, poor angular resolution, and are sensitive to temperature, wind, and sound-absorbing materials [24]. Nevertheless, they continue to play an important role in parking assistance systems, as they provide accurate distance measurement at low speeds. In the future, ultrasonic sensors may increasingly be replaced by short-range radars and terahertz radars, which offer similar or better performance at short ranges but provide more accurate angular resolution and multifunctional usability [48]. In the world of autonomous vehicles, ultrasonic sensors are mainly used to detect the immediate environment. Devices operating in the 20–40 kHz frequency range emit sound waves and then calculate the distance based on the time difference between the emitted and reflected signals. Due to their simple design and low cost, they are an essential component of parking assistance systems. When evaluating them, it is important to consider their range (typically 1–5 m), resolution (a few centimetres), weather effects and environmental noise [7]. Advantages: One of the biggest advantages of ultrasonic sensors is their extremely low price and easy integration: the transducer and electronics are inexpensive to manufacture, and their small size allows them to be hidden in the bumper, rear door or rear-view mirrors. Sound waves are not sensitive to lighting conditions, so ultrasound can be used at night and in tunnels. Due to their simple operation, the sensors have few moving parts and require little maintenance. It is also worth noting that sound waves reflect well even from non-reflective objects, so obstacles made of rubber, textiles or other matt materials can also be detected. Limitations and disadvantages: Ultrasonic sensors are primarily limited by their very short range (1–5 m) and low angular resolution; they are unable to distinguish between multiple objects, and sound waves scatter strongly in lateral directions. The speed of sound depends on temperature, humidity and wind speed, so measurement accuracy can vary; for this reason, systems often include temperature compensation. In addition, external noise sources (engine noise, wind noise, ultrasonic sensors on other vehicles) can cause interference, leading to false alarms. The angle and texture of the reflective surface also affect sound reflection: most of the ultrasound arriving at a steep angle is absorbed or deflected. Areas of application and integration: Ultrasonic sensors are most commonly found in parking assistance systems, where the vehicle is moving at low speed. The driver receives information from the sensors in the form of audible beeps or visual displays; more advanced systems automatically apply the brakes when the ultrasonic sensor detects a nearby obstacle. Ultrasonic technology is also used in the passenger compartment to detect seat occupancy (detecting the presence of children). In future autonomous vehicles, ultrasonic sensors could be used to provide a detailed map of the immediate environment (0–1 m), complementing the longer range of LiDAR and radar. In the context of sensor fusion, ultrasonic signals can contribute to vehicle self-positioning in confined spaces and enclosed areas.

Empirical studies have shown that the accuracy and reliability of ultrasonic sensors lag significantly behind short-range radars, especially in rainy or noisy environments [21,32]. Nevertheless, advanced MEMS-based ultrasonic arrays and ultrasonic-radar integration may offer new possibilities: Appia et al., [49] proposed a low-cost ultrasonic sensor array for vehicle localisation, while Mártinez et al., [50] demonstrated that ultrasound is suitable for passenger occupancy detection and use in occupant monitoring systems. These studies emphasise that ultrasonic technology can remain relevant when combined with innovative integration and processing methods.

Development trends: In addition to traditional piezoelectric transducers, MEMS (micro-electro-mechanical system) ultrasonic sensors have emerged, which operate at low voltages, have high bandwidths, and can be arranged in arrays. MEMS ultrasonic arrays enable electronically controlled beamforming, which can improve angular resolution and increase range. However, advanced arrays increase cost and system complexity. Research is also underway in the field of digital ultrasonic imaging, which involves constructing a sonogram from reflected sound waves, similar to medical ultrasound. However, these technologies are still in the experimental phase and are rarely used in the automotive industry. Critical comments and future challenges: The importance of ultrasonic sensors in the world of autonomous vehicles appears to be declining, as short-range radars and terahertz systems may take over their role in the near future. Radars and LiDARs already provide high-quality data at ranges of up to 10 m and offer significantly better angular resolution and reliability through software focusing or modular antenna arrays. Nevertheless, further development of ultrasonic sensors is important: environmentally friendly and low-energy ultrasonic modules enable vehicles to manoeuvre more accurately and safely in confined spaces. In the future, hybrid systems may emerge in which a combination of MEMS ultrasound, short-range radar and cameras provides a three-dimensional model of the vehicle’s immediate environment. The biggest challenge for ultrasonic systems is to meet the functional safety (ASIL) levels and increased performance expectations required by autonomous vehicles while maintaining low costs [51].

Ultrasonic sensors are primarily used for short-range obstacle detection in autonomous vehicles, particularly in parking assistance and low-speed manoeuvring scenarios. Their typical detection range is limited to approximately 1–5 m, with distance accuracy in the order of a few centimetres. Because of their low cost, small size and simple design, ultrasonic sensors are widely integrated into vehicle bumpers and side panels. Despite these advantages, ultrasonic sensors play a relatively limited role in autonomous driving perception systems compared to cameras, LiDAR and radar. Their short range and limited angular resolution restrict their usefulness in high-speed driving environments. In addition, measurement accuracy may be affected by environmental conditions such as temperature variations, wind and acoustic interference. For these reasons, ultrasonic sensors are typically used as complementary sensors within a multi-sensor architecture rather than as primary perception systems. Their main role is to provide accurate short-distance measurements during parking and low-speed manoeuvres, where high-resolution long-range sensors are less effective.

### 3.9. Inertial Measurement Units (IMUs) and GNSS Systems

The basis for the localisation of autonomous vehicles is the inertial measurement unit, which contains three-axis accelerometers and gyroscopes. The IMU measures the linear acceleration and angular velocity of the vehicle, providing information on six degrees of freedom [13]. As the IMU is based on physical laws, it is independent of the external environment and therefore continues to function even when LiDAR, radar or camera data is affected by rain, fog or dust [13]. By integrating the data over time, the system calculates position and orientation, but continuous integration results in drift, so the IMU alone is inaccurate over long distances. GNSS (Global Navigation Satellite System) systems, which combine GPS, Galileo, GLONASS and BeiDou satellite systems, calculate the absolute position of the vehicle. The DPV Transportation article highlights that GPS signals can be weakened or completely interrupted in urban environments, tunnels or between tall buildings, and multipath reflections can cause inaccuracy [24]. GNSS alone provides metre-level accuracy, which is often insufficient in road environments. The combination of IMU and GNSS sensors (INS inertial navigation system) creates an integrated system capable of high-precision localisation. According to Aceinna’s summary, a vehicle navigation system typically includes a GNSS receiver and an IMU-based INS; when the GNSS signal is interrupted, the INS continues position estimation in “dead reckoning” mode [14]. For accurate localisation, a Kalman filter is often used, which combines GNSS and IMU data with dynamic weighting [14]. With the help of Real-Time Kinematic (RTK) corrections and base stations, position accuracy can be reduced to centimetre level [52].

One challenge of integration is accurate time synchronisation, as GNSS and IMU operate at different sampling frequencies. In addition, sensor bias correction and temperature-dependent drift also require attention. Future developments will integrate additional sensors into navigation systems, such as visual odometry, LiDAR SLAM and wheel speed sensors, which will increase redundancy and robustness. Further improvement is achieved by using RTK corrections, where reference stations measure GNSS errors with centimetre accuracy and correct them in real time [14]. Innovation trends: MEMS IMUs have advanced significantly in recent years: sensor sensitivity and thermal stability have improved, and navchip integrated systems contain a gyroscope, accelerometer, magnetometer and barometer on a single chip. In the field of GNSS, multi-frequency receivers (L1/E1, L2/E5, L5/E6) enable the correction of ionospheric errors and increase accuracy. Protection against jamming and spoofing is improving: adaptive beamforming reduces the impact of interfering signals, and cryptographic authentication guarantees signals from verified sources. Another trend is the supplementation of GNSS signals with V2X-based localisation, where vehicles and infrastructure exchange position information with each other, and the integration of visual odometry and LiDAR SLAM, which results in redundant and robust positioning.

Critical considerations and challenges: When evaluating IMUs and GNSS systems, different quality classes must be taken into account. High-precision laser or fibre optic gyroscopes are expensive and large, while mass-produced MEMS IMUs are prone to drift. GNSS service is also vulnerable due to geopolitical dependencies: the American GPS, European Galileo, Russian GLONASS and Chinese BeiDou have different availability, and signal regulation may be subject to political influence. GNSS signals are easily jammed, so the vehicle must be able to recognise spoofed signals and switch to dead reckoning or visual and LiDAR-based localisation. Accurate time synchronisation is also critical: different sampling frequencies and delays between the IMU and GNSS can lead to errors in fusion algorithms. Finally, sensor calibration and temperature-dependent drift correction require ongoing maintenance. Further technical details: The limitations of inertial systems are often determined by the scale factor, cross-axis couplings, and noise model. White noise, structured (1/f) noise, and bias instability are all sources of error that can be characterised by Allan deviation analysis, which can be used to select the appropriate time window for drift correction. Low-cost MEMS sensors typically have higher white noise and rapidly increasing bias drift, while ring laser or fibre optic gyroscopes are orders of magnitude more stable but many times more expensive. In vehicle dynamics, high accelerations, vibrations and mechanical vibrations introduce additional errors into the IMU output; therefore, mechanical isolation (shock mount), thermal insulation and precise placement of the sensor close to the vehicle’s centre of mass are necessary. Additional sensors and fusion strategies: The IMU and GNSS are not the only components involved in the localisation task: magnetometers use the Earth’s magnetic field as a compass, barometers can provide altitude information, and wheel speed sensors and steering angle sensors measure the longitudinal and lateral movement of the vehicle. Modern autonomous systems also integrate visual odometry: camera- or LiDAR-based simultaneous localisation and mapping (SLAM) uses visual landmarks, characteristic surfaces or dense point clouds to reconstruct motion. This data complements the IMU, reduces drift, and provides redundant control in the event of GNSS signal loss [53]. Sensor fusion algorithms use a mathematical framework for state estimation: the Kalman filter provides an approximate solution for linear systems, the Extended Kalman Filter (EKF) and Unscented Kalman Filter (UKF) for nonlinear systems, while particle filters (PF) use a distribution-based representation that aids in multimodal estimation. Factor graphs and optical smoothing methods optimise in large spatial blocks but delay the result; in real-time applications, a compromise must be found between batch and streaming processing.

Practical implementation issues: In a car, the placement of the IMU and GNSS antennas affects accuracy. The IMU should be mounted near the vehicle’s centre of mass to minimise displacement due to centripetal force, while the GNSS antenna should be placed on the roof, facing the open sky. Cable lengths should be minimised to reduce signal attenuation and phase distortion. Sensor calibration consists of two parts: intrinsic calibration determines the sensor’s own parameters (scale, axis cross), while extrinsic calibration compares the position and orientation to the vehicle’s other sensors. In the case of IMUs, in-field autocalibration is common, which uses certain vehicle manoeuvres (e.g., circular motion, acceleration, deceleration) to determine scale factors and drift. The calibration of GNSS antennas requires knowledge of the phase centre and multi-frequency behaviour. Emerging technologies and research directions: In the near future, new inertial sensors may appear in autonomous vehicles, such as optical integrated gyroscopes, which measure the Sagnac effect in silicon photonic waveguides; quantum sensors, which provide extremely low noise and drift using atomic interferometry; or membrane-vibration MEMS gyroscopes, which achieve high sensitivity even at very low masses. Further development of visual–inertial odometry is also important: neural networks are capable of estimating a combined state vector from raw IMU measurements and a series of camera images, which is more resistant to dynamic disturbances and environmental changes. Machine learning models are now also used to calculate corrective adjustments for IMU drift or to detect early signs of IMU failure. The long-term goal is for the vehicle to be able to self-diagnose the IMU and, if necessary, replace the estimated state with a model derived from other sensors.

Safety, reliability and social dimensions: GNSS jamming or spoofing is not only a technical problem but also a national security issue; the vulnerability of autonomous vehicles can lead to catastrophic accidents if positioning is lost. Therefore, it is recommended to strengthen GNSS protection near critical infrastructure, use multi-constellation receivers, and introduce reconfidence algorithms that verify the GNSS-based position by comparing it with the vehicle’s internal sensors and digital maps. The accuracy of positioning is also important from a social perspective: overly detailed tracking can violate personal data and privacy. Cryptographic protocols need to be developed that authenticate position but preserve anonymity. Future autonomous vehicles are expected to use a hybrid localisation system in which IMU/GNSS, visual and LiDAR odometry, V2X-based position sharing and map-based corrections work together to provide the vehicle’s status.

#### 3.9.1. Sensor Fusion Architectures and Engineering Trade-Offs

**Figure 5:**
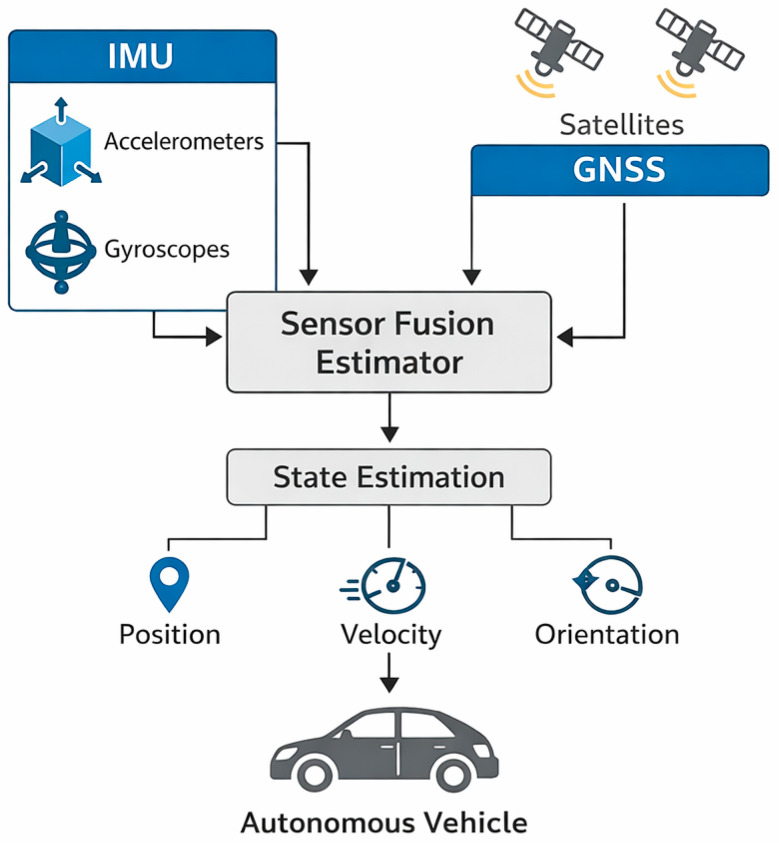
IMU and GNSS cooperation. Source: own edited.

Autonomous vehicles rely on multiple sensors operating on different physical principles, including cameras, LiDAR, radar, ultrasonic sensors and localisation systems. The integration of these heterogeneous data sources is known as sensor fusion. Although many studies highlight the advantages of sensor fusion, its implementation involves significant engineering trade-offs related to architecture, latency, computational requirements and robustness. Three major sensor fusion architectures are commonly distinguished in the literature: early fusion, mid-level fusion and late fusion (Figure 5).

#### 3.9.2. Early Fusion (Data-Level Fusion)

In early fusion architectures, raw sensor data from multiple sensors are combined before feature extraction. For example, LiDAR point clouds and camera images can be projected into a common coordinate system, allowing neural networks to process both modalities simultaneously. The advantage of this approach is that the learning algorithm can discover complex correlations between modalities. However, early fusion requires precise calibration and time synchronisation between sensors. Even small errors in spatial alignment or timestamp differences can lead to incorrect object representations. In addition, raw sensor data streams are extremely large, which significantly increases computational load and memory bandwidth requirements.

#### 3.9.3. Mid-Level Fusion (Feature-Level Fusion)

Mid-level fusion combines features extracted independently from each sensor. For example, convolutional neural networks may first extract visual features from camera images, while separate networks process LiDAR point clouds or radar signals. These intermediate representations are then fused into a unified perception model. This approach reduces data volume compared to early fusion and allows each sensor modality to use specialised feature extraction methods. However, designing compatible feature representations remains challenging. If the extracted features are not well aligned, the fusion stage may fail to combine complementary information effectively.

#### 3.9.4. Late Fusion (Decision-Level Fusion)

Late fusion combines the outputs of independent perception modules. Each sensor produces its own object detection or classification results, and a decision-level algorithm integrates these outputs. This approach is robust against individual sensor failures because the system can still operate if one sensor temporarily becomes unavailable. Late fusion is also easier to implement from a software engineering perspective, as modules can be developed independently. The disadvantage is that potentially useful correlations between raw sensor signals are lost, which may reduce detection accuracy in complex scenarios.

Table 6 illustrates that different fusion architectures involve significant trade-offs between perception accuracy and computational complexity. Transformer-based architectures such as BEVFormer achieve strong performance on large-scale data sets but require substantial GPU resources, whereas simpler fusion methods such as PointPainting offer lower computational cost but may provide less robust multimodal integration.

#### 3.9.5. Engineering Challenges of Real-Time Fusion

Beyond architectural choices, real-world sensor fusion introduces additional challenges. One of the most important issues is time synchronisation. Cameras, LiDARs and radars often operate at different sampling rates (for example, 30 Hz for cameras, 10–20 Hz for LiDAR and up to 100 Hz for radar). If the timestamps of sensor measurements are not aligned, moving objects may appear in inconsistent positions across different sensor modalities. Calibration is another critical problem. Calibration errors can have severe consequences for perception accuracy in autonomous driving systems. Even small misalignments between sensors may produce significant spatial inconsistencies in fused data. For example, a rotational calibration error of only 0.5° between a LiDAR and a camera can result in object projections that are displaced by several metres at distances of 50–100 m. Such errors may cause perception algorithms to incorrectly associate LiDAR points with image features, leading to object detection failures.

Temporal misalignment can produce similar problems. If a LiDAR frame captured at time t is fused with a camera image captured several tens of milliseconds later, moving objects such as pedestrians or vehicles may appear in different positions in the two sensor modalities. In urban traffic scenarios, even a time offset of 50–100 ms can result in position errors exceeding one metre for fast-moving objects. These discrepancies can significantly degrade the performance of multi-sensor perception algorithms. For this reason, modern autonomous vehicle platforms employ continuous calibration procedures and hardware-based synchronisation mechanisms such as GPS-disciplined clocks, precision time protocol (PTP) networks or hardware trigger signals. In addition, online calibration algorithms can dynamically estimate sensor misalignment during operation and compensate for mechanical drift caused by vibration, temperature variation or long-term wear. Accurate sensor fusion requires precise knowledge of the relative position and orientation of sensors mounted on the vehicle. Mechanical vibration, temperature changes and long-term wear can gradually alter sensor alignment, which requires continuous recalibration. Computational complexity also represents a major engineering constraint. High-resolution cameras, dense LiDAR point clouds and radar signal processing all generate massive data streams that must be processed in real time. Efficient fusion therefore requires specialised hardware accelerators, including GPUs, TPUs or dedicated AI chips. Reliability and fault tolerance must be considered. Sensor fusion systems must detect inconsistencies between sensor inputs and isolate faulty measurements. For example, if a camera is blinded by sunlight or a LiDAR signal is degraded by fog, the fusion system should rely more heavily on radar or other sensors. Designing such adaptive fusion strategies remains an active research challenge. These considerations highlight that sensor fusion is not merely a conceptual advantage but a complex system engineering problem involving trade-offs between accuracy, computational cost, robustness and safety.

#### 3.9.6. Improving Reliability Beyond Sensor Redundancy

Although redundancy and multi-sensor fusion are widely used strategies to improve the reliability of autonomous vehicle perception systems, several additional engineering approaches are necessary to achieve the safety levels required for real-world deployment. Modern autonomous vehicle platforms continuously monitor the operational status of their sensors. Built-in diagnostic algorithms analyse signal quality, noise levels, temperature changes and calibration parameters in real time. If a sensor begins to deviate from expected operating conditions, the system can detect the anomaly and trigger corrective actions such as recalibration, sensor reweighting in the fusion algorithm or activation of fallback modes. Reliability can also be improved through modular and fault-tolerant software design. Safety-critical perception and control modules are typically separated from non-critical components and executed in isolated computing environments. Watchdog mechanisms and safety supervisors monitor the execution of software processes and can reset or isolate malfunctioning modules without affecting the entire system. Artificial intelligence models play an increasing role in perception and decision-making, but their behaviour can be difficult to predict. To address this challenge, researchers are developing verification techniques that analyse neural network behaviour under different input conditions. Methods such as adversarial testing, robustness verification and explainable AI aim to ensure that perception algorithms behave consistently even in unusual or rare traffic scenarios.

Sensor performance may degrade over time due to mechanical vibrations, temperature variations or environmental contamination. Continuous calibration algorithms allow autonomous systems to dynamically adjust sensor alignment and internal parameters during operation. In addition, adaptive machine learning methods can improve perception accuracy by updating models based on new driving data collected from different environments. Autonomous vehicle sensors can potentially be affected by malicious interference, such as GPS spoofing, LiDAR signal injection or camera blinding attacks. Reliability therefore depends not only on hardware performance but also on cybersecurity protection mechanisms. Detection algorithms capable of identifying abnormal signal patterns or inconsistent sensor measurements can help prevent such attacks from compromising the perception system.

### 3.10. Extensive Testing and Simulation

Finally, large-scale simulation and scenario-based testing play a crucial role in improving system reliability. Autonomous driving algorithms are increasingly validated in virtual environments that simulate millions of traffic scenarios, including rare edge cases that are difficult to observe during real-world testing. These simulations allow developers to identify potential system weaknesses before deployment. Taken together, these approaches demonstrate that improving the reliability of autonomous vehicle systems requires a combination of hardware redundancy, robust software design, continuous monitoring, cybersecurity protection and extensive validation procedures.

#### 3.10.1. Real-World Autonomous Vehicle Platforms and Sensor Configurations

To better understand the practical implementation of autonomous vehicle sensing systems, it is useful to examine several real-world development platforms used by major companies and research institutions. These platforms demonstrate how different sensor technologies are integrated in practice and illustrate the trade-offs between sensing capability, energy consumption and system complexity (Table 7).

#### 3.10.2. Waymo Autonomous Vehicle Platform

**Figure 6:**
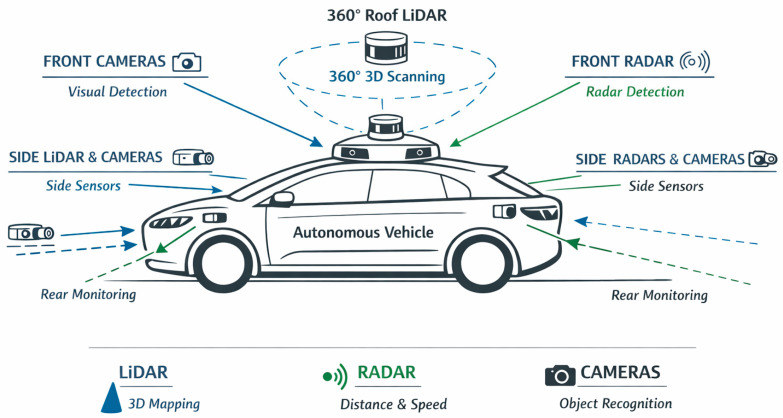
Example of a commercial autonomous vehicle platform (Waymo robotaxi). The vehicle integrates multiple LiDAR sensors, radar modules and cameras to provide full 360° environmental perception.

Waymo, a subsidiary of Alphabet, is one of the most advanced developers of autonomous driving systems. The company operates a fleet of autonomous vehicles based primarily on modified Jaguar I-PACE electric vehicles. The Waymo platform uses a rich sensor suite that includes multiple LiDAR units, cameras and radar sensors distributed around the vehicle (Figure 6).

The main rooftop LiDAR sensor provides a detection range of approximately 300 m and generates a dense 3D point cloud of the environment. Additional short-range LiDAR units are mounted around the vehicle to eliminate blind spots. The vehicle also includes several high-resolution cameras for object recognition and traffic sign detection, as well as radar sensors for reliable operation in rain or fog. Typical specifications of the Waymo platform include an electric driving range of approximately 400 km, a battery capacity of around 90 kWh and a sensor configuration consisting of more than 20 individual sensing modules.

#### 3.10.3. Tesla Full Self-Driving Platform

Tesla approaches autonomous driving using a different philosophy compared with many other companies. Instead of relying heavily on LiDAR sensors, Tesla vehicles primarily use a vision-based perception system supported by radar (in earlier generations) and ultrasonic sensors. A typical Tesla Full Self-Driving (FSD) platform includes eight high-resolution cameras providing a 360-degree field of view, forward-looking radar (in earlier hardware versions), and twelve ultrasonic sensors for short-range obstacle detection. The camera system supports distances up to approximately 250 m for object detection. Tesla vehicles use powerful onboard AI processors capable of performing more than 100 trillion operations per second for real-time neural network inference.

The Tesla Model S and Model Y vehicles equipped with the FSD hardware platform typically have a driving range between 400 and 600 km depending on the battery configuration.

#### 3.10.4. Baidu Apollo Autonomous Platform

The Baidu Apollo autonomous driving platform is widely used in China for robotaxi and research applications. Apollo vehicles typically combine LiDAR, cameras and radar sensors in a multi-sensor architecture designed to maximise redundancy and environmental robustness. Typical Apollo robotaxi vehicles include a 64-beam or 128-beam LiDAR sensor, multiple radar units and up to 12 cameras. The LiDAR sensors usually provide a detection range of 200–250 m, while radar sensors support reliable detection of moving objects in adverse weather conditions. These platforms highlight that practical autonomous vehicle sensing systems rely on heterogeneous sensor configurations and significant computational resources. Differences between platforms also reflect different design philosophies: some systems emphasise LiDAR-based geometric perception, while others prioritise camera-based perception supported by artificial intelligence models.

#### 3.10.5. Safety Comparison Between Autonomous and Conventional Vehicles

An important question related to autonomous driving technology is whether these systems can achieve a safety level that is comparable to or better than that of human-driven vehicles. Road traffic accidents remain one of the leading causes of injury and death worldwide, and studies consistently show that human error is responsible for the majority of accidents. Factors such as distraction, fatigue, alcohol consumption and delayed reaction times significantly contribute to accident occurrence (Table 8).

Autonomous vehicles aim to reduce these risks by replacing human perception and decision-making with sensor-based systems and real-time algorithms. Modern autonomous platforms continuously monitor the environment using multiple sensors, including cameras, LiDAR, radar and ultrasonic sensors. These sensors operate simultaneously and provide redundant perception channels, allowing the system to detect obstacles, pedestrians and other vehicles even when one sensing modality becomes degraded. Statistical comparisons between autonomous and human-driven vehicles are still limited due to the relatively small deployment scale of autonomous systems. However, preliminary studies conducted by companies such as Waymo indicate that autonomous vehicles may achieve lower accident rates in certain scenarios. For example, analyses of several million kilometres of autonomous driving have shown reductions in rear-end collisions and intersection accidents compared with human drivers. Nevertheless, autonomous systems introduce new categories of risk. Unlike human drivers, autonomous vehicles depend on complex software, sensor calibration and data processing pipelines. Failures may occur due to sensor malfunction, incorrect perception by machine learning algorithms or unexpected edge cases in the environment. Furthermore, the interaction between autonomous vehicles and human-driven vehicles can create complex situations that are difficult to model. For this reason, the safety of autonomous vehicles cannot be evaluated solely by accident statistics. It also depends on system architecture, redundancy strategies and functional safety standards such as ISO 26262. Autonomous vehicle platforms typically include multiple layers of safety mechanisms, including redundant sensors, self-diagnostics, fallback control modes and continuous monitoring of sensor health. Overall, while early evidence suggests that autonomous vehicles have the potential to improve road safety, achieving safety levels significantly higher than those of conventional vehicles requires continued development in perception reliability, fail-safe system design and validation under diverse real-world conditions.

#### 3.10.6. Role of Artificial Intelligence in Autonomous Vehicle Development Stages

Artificial intelligence plays a central role in the development and operation of autonomous vehicle sensing systems. Rather than being applied at a single stage, AI technologies are integrated throughout the entire perception and decision-making pipeline of autonomous driving systems. The first major stage where artificial intelligence is applied is environmental perception. Deep neural networks process data from cameras, LiDAR and radar sensors in order to detect and classify objects such as vehicles, pedestrians, cyclists and traffic signs. Convolutional neural networks and transformer-based models are commonly used for image recognition tasks, while specialised neural architectures process LiDAR point clouds or radar signals. Artificial intelligence is also increasingly used in sensor fusion architectures. Instead of combining sensor outputs through purely geometric or probabilistic methods, modern systems use neural networks to integrate heterogeneous sensor data. For example, multimodal neural networks can combine LiDAR point clouds with camera images in bird’s eye view (BEV) representations, allowing the system to create a unified spatial model of the environment.

Another important application of artificial intelligence is the prediction of the future behaviour of surrounding objects. Autonomous vehicles must estimate the likely trajectories of pedestrians, cyclists and other vehicles in order to avoid collisions. Machine learning models analyse past motion patterns and environmental context to predict short-term trajectories. Artificial intelligence can also assist with high-level decision making and trajectory planning. Reinforcement learning and optimisation-based algorithms are used to determine safe driving actions, such as lane changes, braking or acceleration. These algorithms must operate under strict real-time constraints and comply with safety requirements. Finally, AI is used during the development and validation stages of autonomous driving systems. Machine learning techniques help generate realistic simulation scenarios, identify rare edge cases and evaluate system performance across millions of virtual driving situations. This stage is critical because real-world testing alone cannot cover all possible traffic scenarios. Taken together, these stages show that artificial intelligence is not limited to a single component of autonomous vehicles but is embedded throughout the sensing, interpretation, prediction and decision-making pipeline. The integration of AI into these stages significantly improves perception accuracy and system adaptability but also introduces challenges related to safety certification, explainability and computational requirements.

#### 3.10.7. V2X, Vehicle-to-Vehicle, and Vehicle-to-Infrastructure Communication

Vehicle-to-Everything (V2X) communication technologies enable vehicles to exchange information with other vehicles, infrastructure and traffic management systems. Two main technological approaches are currently discussed in the literature: Dedicated Short-Range Communication (DSRC) and Cellular V2X (C-V2X). DSRC is based on IEEE 802.11p wireless communication and was originally designed specifically for low-latency vehicle-to-vehicle communication [54]. C-V2X, in contrast, relies on cellular network technologies defined by the 3GPP standards and can operate both in direct vehicle-to-vehicle mode (PC5 interface) and through cellular infrastructure.

While DSRC was initially considered the dominant approach for cooperative vehicle communication, recent developments suggest increasing industry interest in C-V2X solutions due to their compatibility with existing mobile networks and potential integration with future 5G and 6G communication infrastructures. However, both technologies aim to support safety-critical applications such as collision warnings, cooperative perception and traffic coordination. Detailed technological differences: The differences between DSRC and C-V2X lie not only in the physical layer used but also in the business model and regulatory environment. DSRC operates as a self-organising network where vehicles communicate directly with each other via the IEEE 802.11p framework; this technology has been around for decades and has been deployed on thousands of kilometres of road as a test infrastructure. C-V2X, on the other hand, is based on the 3GPP Release 14/16 standards and has two modes: mode 3 (network-assisted), where the mobile network allocates traffic resources, and mode 4 (network-independent), where vehicles directly coordinate frequency bands. The main advantage of C-V2X is the reuse of LTE/5G infrastructure and greater range, but it increases network load and roaming complexity between operators.

Regulatory and market situation: Different regions have different attitudes towards V2X technologies. In 2020, the US Federal Communications Commission (FCC) decided to allocate the 5.9 GHz band, reserving two-thirds of it for C-V2X, thus effectively targeting the suppression of DSRC. In Europe, the ETSI standardisation body continues to support the ITS-G5 (DSRC) solution, while C-V2X from 3GPP is also permitted; the coexistence of the two systems raises questions about interoperability and costs. In China, C-V2X systems receive greater support due to the strong presence of mobile network operators and consistent government regulation. These regional differences make it difficult for global fleet operators to develop a unified vehicle platform.

In-depth applications and use cases: Key applications for V2X include situational awareness collision avoidance, where vehicles continuously share their position, speed and direction to anticipate dangerous situations; remote driving and teleoperation, where a human operator can intervene in the control of a vehicle via the network; cooperative adaptive cruise control (CACC) and platooning, where vehicles travel in convoy to reduce air resistance and energy consumption; and cooperative perception, which allows vehicles to share sensor data (e.g., LiDAR point cloud) and jointly “see” around corners. In future systems, intersection control units (ICUs) will coordinate vehicle passage in real time, minimising congestion and the chance of collisions.

Network architectures and the 6G perspective: The network architecture of C-V2X and future 6G systems is based on the concepts of edge computing and network slicing. Edge computing allows sensor data to be processed close to the cell tower, reducing latency, while network slicing ensures that safety messages travel on a dedicated, reliable channel. 6G research is exploring technologies such as terahertz communication, reconfigurable intelligent surfaces (RIS) and cell-free massive MIMO, which could provide even higher data speeds and wider coverage. These developments will enable vehicles to share raw point clouds, thermal camera images and radar spectra with each other in real time, radically improving cooperative perception [55].

Cybersecurity and data protection in detail: Due to the openness of V2X networks, malicious attacks pose a real threat. Public Key Infrastructure (PKI) systems oversee identity authentication; vehicles use certificates to sign messages, but scaling certificate management for millions of vehicles is a challenge. The misbehaviour detection system (MDS) monitors message traffic and detects anomalies (false positions, unusual message flow). MDSs (pseudonym certificates) ensure that vehicles can be identified in the event of malicious activity but that data cannot be traced back to the owner in everyday traffic. User confidence is increased by clearly communicating that V2X messages do not contain personal data and that the network does not track the location of vehicles over the long term. Social and economic considerations: The deployment of V2X systems requires significant investment from both vehicle manufacturers and governments. The installation of DSRC-based roadside units could cost billions of dollars, while C-V2X-based systems use the infrastructure of mobile network operators but may involve high subscription fees. Consumer acceptance will depend largely on whether they perceive direct benefits (e.g., fewer accidents, faster travel). Legal issues, such as who is liable for a collision if the vehicle made a decision based on a V2X message, have not yet been clearly resolved. V2X systems will primarily become widespread when, in addition to safety benefits, economic incentives (e.g., lower insurance premiums) also emerge [56].

Critical reflections: Although V2X has the potential to revolutionise autonomous transport, it cannot be considered a solution to all problems. Due to the uncertainty of wireless communication, limited frequency bands and network latency, V2X can only provide supplementary information and can never replace on-board vehicle sensors. Economic life cycle analyses show that V2X investments will only pay off if they significantly reduce the number of accidents and achieve significant fuel savings through traffic optimisation. Organisations must be careful not to create a new digital divide between urban areas with advanced infrastructure and rural regions. In addition, the rights of vehicle owners (e.g., the option to refuse software updates) must also be respected.

V2X (Vehicle-to-Everything) communication systems extend the field of vision of autonomous vehicles by transmitting information beyond that provided by sensors, such as the status of traffic lights, warnings about accident situations or the movements of other vehicles. Current V2X technologies can be divided into two main groups: Dedicated Short-Range Communications (DSRC), which is based on the IEEE 802.11p protocol and provides direct, low-latency (≈2 ms) data exchange over short distances; and Cellular V2X (C-V2X), which enables vehicle-to-vehicle, vehicle-to-infrastructure, vehicle-to-pedestrian and vehicle-to-network communication via LTE and 5G networks [1].

**Figure 7:**
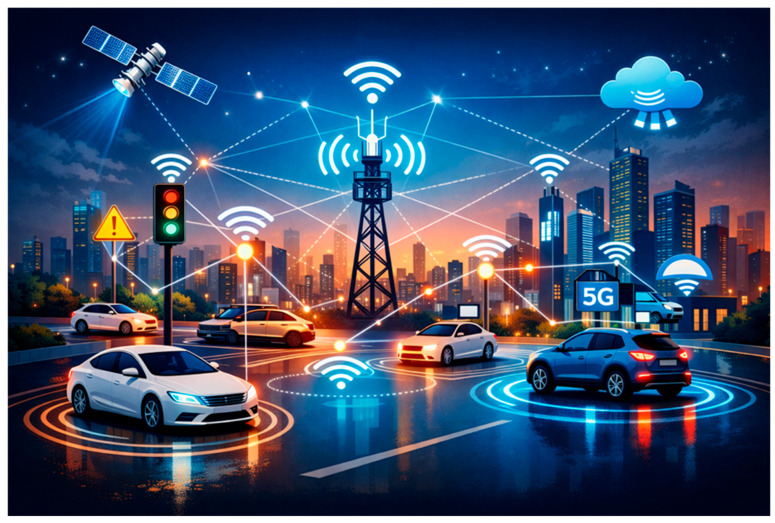
V2X communication network. Source: own edited.

Advantages and applications: The biggest advantage of V2X is that it provides information beyond the line of sight: the vehicle can know in advance the status of an intersection or receive notification of an approaching ambulance even before it comes into view of the sensors. The low latency of DSRC systems is particularly useful for critical safety functions such as forward collision warning and lane departure warnings. C-V2X systems offer wider coverage as they use mobile network infrastructure, enabling real-time mapping services, traffic optimisation and fleet management. With the spread of 5G and 6G networks, the bandwidth and reliability of V2X systems will continue to increase, enabling new applications such as platooning and cooperative perception. Limitations and challenges: DSRC systems have the disadvantage of limited range (300–500 m) and the need for dedicated infrastructure, while C-V2X systems are dependent on mobile network operators and network congestion. Latency can increase due to network load and cell handover, which can pose a risk for critical safety messages (Figure 7). Common standards and global frequency allocation are essential for the success of V2X systems; regional differences (US, EU, China) make global vehicle interoperability difficult. Cyber security is of paramount importance: the lack of communication encryption, certificate management and anonymity undermines confidence in the entire system. Spoofing or denial-of-service (DoS) attacks pose a significant accident risk. Technological trends and integration: In the future, V2X systems will continue to evolve in parallel with 5G and 6G networks. Network slicing and edge computing will enable dedicated network slices for low-latency safety messages. C-V2X Mode 4 enables direct vehicle-to-vehicle communication independently of the network, which increases reliability in rural areas. Research aims to combine URA (Ultra-Reliable and Low Latency Communications) and mMTC (massive Machine-Type Communications) to enable vehicles to share raw sensor data (e.g., LiDAR point clouds) at data rates of several gigabits per second and make decisions together (cooperative perception). The integration of V2X systems into sensor fusion poses a new challenge: how can the vehicle’s own sensor data be combined with information received from the network with uncertain timestamps? Time synchronisation, data compression and relevance-based filtering are key areas of research [57].

Critical considerations: Although V2X systems are promising, the introduction of the technology is not without criticism. Competition between DSRC and C-V2X is dividing the industry, and some countries (e.g., the US) have already decided in favour of C-V2X, while other regions are still building DSRC infrastructure. The dual solution causes compatibility issues and increases vehicle costs. Furthermore, data protection and personal data management raise serious ethical questions: the data shared by vehicles could even be used to track passengers’ habits. V2X systems must therefore be reliable not only from a technical point of view but also from a legal and social perspective. Finally, it is important to note that V2X cannot replace on-board sensors: it merely supplements them and provides redundant information. The autonomous vehicles of the future will likely combine a variety of communication and sensing technologies to ensure safe and ethical operation in all circumstances [58].

#### 3.10.8. Environmental Effects and Sensor Vulnerabilities

##### Precipitation, Fog and Visibility

The sensors in autonomous vehicles respond differently to weather conditions. According to the MDPI review article, LiDAR, radar, camera and ultrasonic sensors are all sensitive to precipitation but to varying degrees [3]. For LiDAR systems with wavelengths of 905 nm and 1550 nm, Mie scattering reduces signal intensity in rain, while extinction coefficients increase dramatically in fog. The effect of rain is demonstrated by the fact that in 2 mm/h rain, the visibility range of LiDAR can decrease from 2 km to 1.2 km, and at an intensity of 25 mm/h, the signal threshold deteriorates even further [11]. In radars, rain noise caused by short-term precipitation can increase the number of false alarms, but the decrease in energy frequency density is only significant over long ranges [11]. In cameras, water droplets, fog and snow distort the image; refraction and scattering reduce contrast. Solutions include hydrophobic coatings, lens heating and real-time image processing algorithms such as dehazing and deraining neural networks. The frequency of ultrasonic sensors is less affected by precipitation, but a film of water on the sensor surface can dampen sound waves.

Detailed modelling of weather effects is being investigated in a number of studies: these include changes in LiDAR reflection coefficients [23,34], adaptive filtering of radar rain noise [40], weather-dependent changes in V2X channel conditions [57], and adaptive scheduling of weather sensor management [59]. The studies emphasise that autonomous vehicles must adapt not only their sensor hardware but also their scheduling and processing strategies to the weather.

#### 3.10.9. Temperature and Extreme Conditions

The operation of sensors is temperature-dependent. For example, IMU gyroscopes and accelerometers have temperature-dependent bias, so thermal compensation is required for precise integration. The laser diodes in LiDAR systems reduce performance at high temperatures, while frost can prevent the lens from rotating. In the case of radar antennas, thermal fluctuations can affect impedance and modulation accuracy. The heated cover of thermal cameras helps to protect against icing [7].

#### 3.10.10. Lightning and Electromagnetic Interference

The MDPI article lists the direct and indirect effects of lightning strikes on sensors: LiDAR and radar are subject to high electromagnetic interference, while GNSS systems can also experience electromagnetic and electrical interference. Direct effects of lightning strikes include circuit damage; indirect effects include errors caused by nearby induction and static charges. The grounding and shielding of electronic systems is key to protecting against these effects [60].

#### 3.10.11. Sensor Fusion and Data Processing

No single sensor is capable of providing a complete and reliable perception of the environment on its own. LiDAR provides accurate 3D point clouds but is expensive and sensitive to weather conditions; radar reliably measures speed in poor weather conditions, but has poor resolution; cameras provide rich colour information but are sensitive to lighting conditions; and IMUs are suitable for measuring motion but drift over time [57]. The aim of sensor fusion is to combine complementary information from different sensors, ensure redundancy and reduce errors (Table 9).

The following advantages are particularly important:

- Redundancy and fault tolerance: if one sensor fails or its quality deteriorates, the other sensors can compensate; this is essential for ensuring functional safety.
- Increased accuracy: reduction of measurement uncertainties in sensors; for example, the combination of LiDAR and camera allows 3D point clouds to be coloured.
- Robust decision-making: artificial intelligence systems that integrate data from different sources make more accurate and faster decisions. Systems equipped with sensor fusion can reduce response times to less than 100 ms, which reduces the likelihood of accidents.
- New functionalities: for example, using radar speed information to refine LiDAR point clouds, or integrating V2X data into the environmental map.

#### 3.10.12. Fusion Levels and Algorithms

Sensor fusion can be implemented at several levels of abstraction:

Low-level fusion: the time- and space-synchronised outputs of the sensors are projected onto a common coordinate system and then integrated. For example, after calibrating the LiDAR point cloud and the camera image, the points are projected onto the image, which allows the creation of a coloured 3D map. The advantage is maximisation of information, but it requires high bandwidth and computing capacity. Feature-level fusion: first, features are extracted from each sensor and then combined. For example, the features generated by the camera CNN are merged with the convolutional features of the LiDAR voxel grid and then fed into a common network.

**Figure 8:**
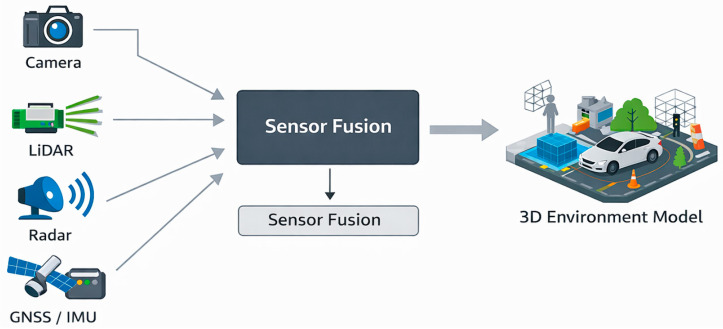
Sensor fusion architecture. Source: own edited.

Decision-level fusion: each sensor has a separate decision-making module that identifies objects, and then the decisions (hypotheses) are combined (e.g., Bayesian networks, rule-based systems). Modern systems often use deep neural networks for fusion. Bird’s eye view (BEV) representations project the 3D point cloud onto a 2D grid and align it with the camera characteristics. Transformer-based fusion models (e.g., BEVFormer) use a multi-head attention mechanism to explore correlations between sensors. Such systems require high data processing capacity but are extremely accurate. The basis for the efficiency of fusion is precise sensor calibration and time synchronisation. Different sensors operate at different sampling frequencies and with different delays; without accurate time stamps, fusion will be inaccurate. Calibration consists of three components: (1) intrinsic calibration, which determines the internal parameters of each sensor (e.g., focal length, distortion); (2) extrinsic calibration, which determines the relative position and orientation of the sensors; (3) time calibration, which corrects for delays. Real-time systems often use time-division multiplexing (TDM) or hardware trigger systems to ensure that the sensors sense simultaneously. The diagram below shows a general sensor fusion architecture. Data streams from the camera, LiDAR, radar and GNSS/IMU sensors arrive at a central fusion module, which evaluates and combines the different sources. The result is a unified environmental model that is used by decision-making algorithms (Figure 8).

#### 3.10.13. Functional Safety and Reliability

##### ISO 26262 and the Automotive Safety Integrity Level (ASIL)

Safety is paramount when designing autonomous vehicle systems. The ISO 26262 standard is the international standard for functional safety in the automotive industry, covering the life cycle of electronic and electrical systems. The standard introduces the concept of Automotive Safety Integrity Level (ASIL), which defines four levels (A–D) based on the risks of the system. The levels are determined by a combination of three factors: (1) the severity of injury resulting from a fault, (2) the probability of a fault occurring, and (3) the controllability of the fault, i.e., the extent to which the driver or the system can avert the danger [59]. ASIL D is the highest level, which is required for systems designed to prevent serious injury or fatal accidents (e.g., braking systems, steering) [60].

The standard requires hazard analysis and risk assessment (HARA) to be carried out, in which developers identify all potential hazards and determine the associated ASILs. In the case of sensors, functional safety objectives mean that the sensors must operate without error or switch to a safe mode in the event of a malfunction (fail-safe/fail-operational). For example, in the event of overheating or lens contamination, the LiDAR module must be able to maintain performance with a self-cleaning function or notify the system of the fault. In autonomous systems, redundancy is key to fault tolerance. Multiple copies of critical sensors must be used (e.g., two radars, multiple cameras) so that the failure of one sensor does not cause a complete system failure. Diversified redundancy means combining sensors with different operating principles, which reduces common cause failures. For example, the combination of terahertz radar and LiDAR provides more reliable information in both bad weather and environmental shadows. Sensors and V2X communication provide a network connection, which is a potential attack surface. Vehicles must be able to withstand man-in-the-middle and spoofing attacks, which can lead to incorrect decisions by manipulating sensor outputs. Root of trust elements, encrypted communication and authentication mechanisms must be integrated into the system design. The ISO/SAE 21434 standard [60] focuses specifically on automotive cybersecurity and is to be used in conjunction with ISO 26262 [36,61].

Terahertz (THz) radar is not only part of the current technological race but also opens up new areas of application. The internal structure of THz radar is similar to that of cameras: the antenna array scans the environment row by row, and the returning signals are used to create a point cloud without moving parts [10]. The technology is expensive, but thanks to Moore’s Law, the production of terahertz transistors is becoming cheaper, and several car manufacturers will be testing the solution by 2028 [9]. Another interesting area is the use of metamaterial sensors [47]. Metamaterials are artificial structures with special electromagnetic properties (negative refractive index, directional transmission). These enable the creation of miniaturised, high-sensitivity antenna surfaces, which are used to optimise radar systems or V2X antennas [62]. Metamaterial-based radar sensors improve angular resolution by increasing bandwidth and antenna array width while reducing noise levels. Optical quantum sensors are based on the quantum state of photons, which are particularly sensitive to small changes in the environment. The concept of quantum LiDAR, for example, uses quantum entanglement to reduce noise levels and increase signal transmission efficiency, making it reliable even in low light conditions. This technology is currently in the research phase, but in the future, it could enable vehicles to detect objects at long distances and even in light-polluted urban environments [63,64].

Photonic processors are a new paradigm in signal processing: they use light to perform calculations. The huge data streams generated by LiDAR and camera systems can be processed by photonic neural networks, which are orders of magnitude faster and more energy-efficient than traditional electronic processors. In 2025, researchers at Harvard and MIT unveiled a photonic chip that performs convolutional operations using light interference, paving the way for the automotive application of optical neural networks. The development of sensors and artificial intelligence does not depend solely on hardware. Digital twin models of autonomous vehicles allow sensors to be tested and validated in a virtual environment. The digital twin is a realistic replica of the vehicle’s physical and software components; it synchronises the vehicle’s status in real time with the model running in the cloud. This allows developers to simulate dangerous situations without putting the physical system at risk [62]. With data-driven simulations, even rare accident situations can be generated in large numbers, which improves the generalisation ability of machine learning models. In the future of autonomous vehicles, intelligent systems that dynamically modify the operation of sensors will emerge. The essence of adaptive sensor management is to modify sensor parameters (e.g., LiDAR pulse density, radar bandwidth, camera exposure time) depending on environmental conditions and the task at hand and to make decisions about which sensors to rely on. Artificial intelligence learns sensor scheduling algorithms that also optimise the system’s energy management. For example, in good visibility conditions, the system can send fewer LiDAR pulses, while increasing radar activity in foggy conditions [65,66].

The development of sensors for autonomous vehicles raises ethical and legal questions. Who is responsible for an accident caused by faulty sensor fusion or incorrect V2X information? How can privacy be ensured when vehicle cameras continuously record the environment and the data is analysed via cloud-based services? It is up to policymakers to develop appropriate regulations that promote innovation while protecting individual rights and public safety [67].

#### 3.10.14. Discussion and Critical Analysis

The complex ecosystem of sensors in self-driving vehicles involves numerous technical and economic compromises. One sensor will never be enough: high-resolution LiDAR is too expensive and weather-sensitive, radar is cheap and robust but less detailed, and cameras provide abundant visual information but are easily blinded. Sensor fusion is therefore not optional, but mandatory. At the same time, calibration, handling large amounts of data, time synchronisation and real-time processing pose significant challenges in integrated systems. The advent of solid-state LiDAR and FMCW technology reduces cost and increases reliability, but high-frequency radars and terahertz sensors will also be serious competitors. Cost and size: The price of sensors is a critical factor for vehicle manufacturers. While dozens of sensors can be used in the premium segment, cost constraints in mass production require compromises. Terahertz radar is currently expensive, but within a few years, the price may be competitive with LiDAR. Cameras are inexpensive, but multiple cameras are needed for redundancy. Ultrasonic sensors are inexpensive, but their functionality is limited. Robustness and weather: Extreme weather conditions pose the greatest challenge. Future systems must include sensors that can operate in bad weather (e.g., terahertz radar, thermal cameras). Redundancy and adaptive management strategies can help avoid errors, but maintenance costs and energy consumption increase. Data processing and artificial intelligence: The amount and diversity of data generated by sensors is growing rapidly. In the future, photonic and neuromorphic processors may reduce response time and energy requirements, but the development of software algorithms is at least as important. Deep learning models must function reliably under changing conditions and avoid errors caused by bias and incomplete training data. Regulation and social acceptance: The sensors in autonomous vehicles raise data protection and ethical concerns. V2X communication and camera-based surveillance present potential opportunities for abuse. Without transparent data management, encryption, and adherence to ethical guidelines, social trust could be undermined. The development of sensors for self-driving cars is an extremely dynamic field. In the coming years, terahertz radar, FMCW LiDAR, neural network-assisted radar, and photonic sensors could bring about the biggest breakthroughs. However, classic sensors (cameras, radar, LiDAR, IMU) will continue to form the backbone of the system, and sensor fusion will improve vehicle safety with increasingly sophisticated methods.

## 4. Discussion

The road to autonomous vehicles is paved with the harmonisation of a complex ecosystem of sensors and data processing. In this study, we reviewed in detail visual sensors (RGB, HDR, event and thermal cameras), different types of LiDAR systems (mechanical, solid-state, FMCW), the evolution of radars from millimetre-wave systems to terahertz devices, ultrasonic sensors, IMU/GNSS localisation and V2X communication networks. In our analysis, we discussed environmental effects, functional safety, and sensor fusion techniques and evaluated the advantages and disadvantages of each sensor type at length. Below, we summarise the most important findings and critical observations and outline future research directions.

First, we emphasise the importance of sensor complementarity. No single sensor is capable of fully interpreting the real world on its own: RGB cameras provide rich colour and texture information but are sensitive to lighting conditions; LiDAR provides centimetre-accurate distance measurement but its performance deteriorates in fog and rain [11]; radar works reliably in all weather conditions, but its low resolution limits its classification capabilities; ultrasound is cheap and simple but can only be used within a range of a few metres; and the combination of IMU and GNSS provides absolute position and direction, but accumulates drift and signal loss may occur [59]. The purpose of sensor fusion is to compensate for these shortcomings: combining sensor data increases robustness, reduces uncertainty and provides redundancy [4]. From a critical point of view, it is important that the implementation of fusion progresses not only at the hardware level but also at the algorithmic level; the time synchronisation, spatial registration and joint representation of data from different sensors is a complex task that determines the response time and reliability of the system.

A detailed discussion of environmental effects has highlighted that weather and environmental factors such as rain, fog, snow, glare, temperature extremes, and electromagnetic interference have a complex influence on sensor performance. The performance of LiDAR and camera systems is particularly sensitive to Mie scattering and refraction, which significantly reduce range and image contrast [11]. Radars are more tolerant of precipitation, but rain noise can cause false alarms [11]. Ultrasound and IMU/GNSS systems are relatively independent of visual conditions but have their own temperature and noise dependencies. Terahertz radars and thermal cameras offer new possibilities for overcoming extreme weather conditions but are currently expensive and have limited availability [60]. To achieve weather-independent sensing, researchers are developing adaptive systems that can select the most reliable sensors in real time based on current conditions and dynamically optimise sensor power consumption.

The data processing and computational requirements of sensors are becoming an increasingly important consideration. While cameras and LiDAR systems generate gigabytes of data, radar and ultrasonic sensors generate much smaller volumes of data but require complex signal processing. Convolutional neural networks, transformer-based models and graph neural networks achieve incredible accuracy in object recognition, but they have high energy requirements and currently can only run on high-performance GPUs or specialised AI accelerators. In the future, edge AI and neuromorphic processors may enable real-time processing with low power consumption, but these are still in their early stages. The reliability of software algorithms is critical; existing models often learn from biassed data sets, leading to over-optimism in real-world environments. In the future, more diversified data sets containing real-world conditions will be needed, including regional differences, variable weather and different infrastructures.

Functional safety and regulation is another key area. The ISO 26262 standard and ASIL classification ensure that electronic systems meet safety requirements; sensors must provide self-checking, redundancy and failure mode recognition. Regulations for V2X communication (DSRC vs. C-V2X) vary from region to region, which poses a challenge for the global automotive industry. Protection against cyber security risks hacking, denial of service, and false messages is just as important as physical sensor protection. Ethical and data protection considerations are also coming to the fore: data collected by cameras and V2X systems may contain sensitive information that can only be protected with appropriate encryption and data management protocols.

Economic considerations cannot be ignored either. Although the price of LiDAR systems is falling, it is still high; radars are cheaper, but 4D imaging radars may push up the cost again. Cameras are relatively inexpensive, but multiple units are required for redundancy. Thermal and event cameras are currently expensive, but prices are expected to fall as production volumes increase. Ultrasonic sensors are very inexpensive but have limited functionality; they are expected to fade into the background as the market shifts. The choice of sensor therefore depends on the target market and vehicle category: luxury cars can integrate more and more expensive sensors, while cost-sensitive solutions are becoming more widespread in mass-produced vehicles. In the future, scalable modular architectures will allow users to choose the sensor configuration based on the desired level of safety and price.

Artificial intelligence and machine learning play a key role in the sensor systems of autonomous vehicles. Neural networks can control not only object recognition but also sensor fusion, localisation, route planning and vehicle control. In radar systems, AI helps to produce super-resolution images and filter out false targets, while deep learning networks can learn state-dependent priors from LiDAR and camera data. The biggest challenge, however, is reliability: the operation of neural networks is often a black box; explainability of decisions, safety certification and real-time fault detection are currently active areas of research. Quantum machine learning and federated learning offer new possibilities, but their practical application is still a long way off. Several sensor technologies of the future stand out. FMCW LiDAR enables speed measurement in addition to distance and can be integrated with CMOS processes, which could lead to lower prices [8]. Terahertz radar has enormous potential due to its extreme resolution and weather resistance, provided that manufacturing and spectrum regulation issues are resolved [9,10]. Neuromorphic event cameras could revolutionise image sensing with their incredible temporal resolution [6], and quantum and metamaterial sensors could bring radically new capabilities in the long term. However, these technologies are not yet mature; their introduction will require new processing hardware, standards and validation methods.

V2X communication is another pillar of sensor horizon extension. Low-latency DSRC and LTE/5G-based C-V2X systems enable vehicles to share information beyond their line of sight, thereby increasing safety and energy efficiency [1]. The latest reviews [68] comprehensively discuss the security, privacy and standardisation challenges of V2X systems, highlighting compatibility issues between DSRC and C-V2X, the importance of authentication and encryption, and the need for global harmonisation of the frequency bands. Cooperative perception and platooning require reliable network infrastructure, standardised message formats and strong cybersecurity. Critically, the rollout of V2X systems is slow in many countries; competition between DSRC and C-V2X, frequency allocation and data provider business models are all hindering rapid adoption. Furthermore, without privacy and anonymity, user confidence may falter.

Ethical and social issues permeate every aspect of autonomous vehicles. Cameras and V2X systems can collect personal data; artificial intelligence decisions affect human lives; and the manufacture of sensors requires raw materials and has an environmental impact. Before autonomous vehicles become widespread, there needs to be a broad social dialogue about what compromises society is willing to accept between safety, data protection and freedom. Ethical algorithms, auditable decision-making logic and transparent risk management are essential. It is particularly important to clarify liability issues in the event of accidents: who is liable if the sensors provide incorrect data or the fusion algorithm makes a wrong decision? The future of autonomous vehicles raises not only technical but also legal and philosophical questions.

The reliability of sensors is closely linked to operational safety and user confidence. Instrument errors, ageing and environmental influences (e.g., temperature fluctuations, mechanical stress, contamination) can lead to sensor degradation over time. Reliability tests must therefore include accelerated ageing tests, vibration and temperature cycle loads. Built-in self-test functions, redundant sensors and diagnostic algorithms help to detect faults before they compromise the safety of the system. The ISO 26262 standard requires the concept of “intrinsic safety”, i.e., that sensor failure must not lead to a dangerous situation; this includes fault detection, degraded mode and emergency mode.

The production and supply chain perspective is of paramount importance for the future of sensor technologies. The manufacture of LiDAR, radar and camera modules requires specialised semiconductor, optical and mechanical components that rely on a global supply chain. The COVID-19 pandemic and geopolitical tensions have highlighted the risk of chip shortages: many manufacturers in the automotive industry have been forced to reduce production due to a lack of sensor components. The growing demand for rare earth metals (e.g., gallium, indium) and silicon carbide is causing logistical and environmental problems. The challenges for the future are the localisation of sensor technologies, the search for alternative materials and the introduction of waste-reducing manufacturing methods (e.g., additive manufacturing). In line with the circular economy, strategies for recycling, taking back and refurbishing sensors also need to be developed.

Social acceptance and usability are also key. Users are often distrustful of autonomous systems, especially when the vehicle is “blinded” in bad weather or traffic signs are poorly maintained. When designing the human–machine interface (HMI), consideration must be given to how sensor errors can be communicated to the driver: for example, a sensor error message should be understandable but not cause panic. Different cultural attitudes and regulations in different countries influence the acceptance of autonomous technologies; developers must adapt functions such as driver intervention strategies accordingly.

There is a cost–performance trade-off for all sensor types. Mechanical LiDAR is significantly more expensive than cameras but offers high accuracy in return. The advent of solid-state LiDAR reduces costs, but its performance does not yet match that of the mechanical version in all areas. Radar is inexpensive and robust, but 4D imaging versions are currently expensive. Ultrasound is inexpensive, but its functionality is limited. It is up to car manufacturers to design modular sensor equipment that is scalable: low-cost models use fewer sensors, while premium vehicles can integrate more advanced sensors. In the future, software updates will play an increasingly important role: sensor hardware is durable, but software is constantly evolving, providing new features and security fixes.

Without accurate sensor calibration, even the best sensors can provide incorrect data. Calibrating the relative position and orientation of cameras, LiDAR and radars (extrinsic calibration) is complex, especially when the vehicle body is deformed or the sensors are subject to vibrations. Calibration errors can distort the results of fusion algorithms. In the future, real-time, self-calibrating algorithms will be needed that infer sensor displacement based on the dynamic behaviour of the vehicle and the objects detected. In addition, accurate timing is important: the clocks of the sensors and the processing system must be synchronised for data fusion to be correct. IEEE 1588 Precision Time Protocol (PTP) and ISO 26262 compliance can ensure the necessary accuracy [69].

Research trends are increasingly pointing towards system-level integration. Sensors alone are not sufficient; autonomous driving also requires high-resolution maps, V2X networks, cloud services and roadside infrastructure. Cooperative perception allows multiple vehicles to share sensor data with each other, enabling the detection of obstacles and accident-prone situations that are beyond the horizon. Digital twins and simulation environments aid safety testing; data-driven simulations model real traffic situations, which is also suitable for recognising rare and dangerous events. Researchers are developing new sensor fusion algorithms (e.g., graph-based and topological methods) and designing architectures optimised for energy efficiency.

Without harmonisation of global standards and regulatory frameworks, the widespread introduction of autonomous technology cannot be achieved. Compatibility between DSRC and C-V2X, radar spectrum allocation, LiDAR safety standards (eye damage limits) and data protection guidelines are all areas where international cooperation is needed. Standards can help to standardise the supply chain, reduce development costs and accelerate the spread of innovation. At the same time, overly strict regulation can stifle creative solutions, so a flexible framework is needed to support research and responsible experimentation.

Finally, it is important to note that the development of sensors for autonomous vehicles will be a long-term, iterative process. The technologies available today, however impressive, are not yet sufficient for completely human-free driving. In the coming years, we are likely to see hybrid systems: partially autonomous vehicles that are capable of operating independently in specific environments (e.g., motorways, closed industrial parks), while still requiring human supervision in urban environments. The biggest challenge for developers is to achieve scalability, interoperability and user confidence simultaneously. This requires not only technological innovation but also social and legal innovation.

Finally, environmental and sustainability considerations must also be taken into account. The manufacture of sensors and processors is energy- and material-intensive, and the mining of rare earth metals can cause environmental damage. The weight and energy consumption of vehicles may increase due to the large number of sensors, which is contrary to climate protection goals. Design considerations must take into account the recyclability of sensors, the principles of the circular economy, and the minimisation of the carbon footprint of the ‘ ’. The vehicles of the future may use fewer but smarter sensors and rely more on networked cooperation and smart infrastructure. Equipping autonomous vehicles with sensors is a multidisciplinary challenge involving photonics, microwave technology, computer science, control engineering, ethical law, and economics. As things stand, no single type of sensor is capable of ensuring safe autonomous driving on its own; the key lies in the combined use of sensor fusion, artificial intelligence and communication networks. The task for future researchers is to increase the reliability of sensors, reduce prices, optimise energy consumption and strengthen social acceptance. Only by coordinating solutions to these complex tasks can the vision of autonomous vehicles making transport safer, greener and more liveable become a reality.

### Extended Conclusions and Vision for the Future

When examining the sensor systems of autonomous vehicles, it becomes increasingly clear that technological development is only one part of the puzzle; environmental, social and economic factors are equally important. In this subchapter, we expand on the previous conclusions, critically interpret the current state of affairs, and formulate our recommendations for future research and regulation.

Complementarity and holistic design: One of the most important lessons is that the complementarity of sensors is not limited to basic functions (distance measurement, image capture). Different sensor types operate in different information spaces: radar measures speed in the Doppler range, LiDAR provides a 3D point cloud, the camera provides 2D visual texture, and the IMU provides motion. An efficient autonomous system builds a common representation from these, assigning adaptive weights depending on environmental conditions. This requires vehicle- and infrastructure-level system design, where instead of optimising individual modules, the energy, cost and reliability profiles of the entire perception chain are examined.

Data quality and fair systems: The quality of sensor data is not uniform; camera images taken in low light, LiDAR point clouds collected in the rain, and noisy radar signals can all distort perception. AI-based algorithms often struggle with bias issues: for example, pedestrian detection models may perform worse for people with dark skin if the training data is not representative. When designing future autonomous systems, fairness criteria must be taken into account: sensors and algorithms must be evaluated under different demographic and environmental conditions, and it must be ensured that no single group is disadvantaged. Data diversification (using data from different regions, seasons, and times) and benchmarking are essential for robust models.

Sustainability and environmental impacts: The manufacture of sensors requires rare earth metals, gallium, indium and other critically scarce materials. Extraction often causes environmental damage and is associated with social problems (e.g., labour abuses). The increase in the number of autonomous vehicles due to sensors, computers and cooling systems may increase energy consumption, which is contrary to climate protection goals. It is therefore important to apply the principles of the circular economy: modular design of sensors, use of recyclable materials, and minimisation of production waste. Life cycle assessment (LCA) methods should be used to evaluate the entire life cycle of sensors, from mining to use to waste management, and can provide guidance to decision-makers on choosing sustainable technologies.

Global standards and cooperation: The success of autonomous vehicles depends largely on global harmonisation. Currently, radar frequency ranges, V2X communication bands, safety regulations and data protection guidelines are regulated differently in each region. This not only makes product development more expensive but also hinders the spread of the technology. International bodies such as ISO, IEEE, 3GPP and UN EGB could develop uniform guidelines that respect regional characteristics but avoid fragmentation. Open standards and open-source software allow smaller players to participate in innovation and reduce the likelihood of monopolies forming.

Research directions and technological innovation: The sensor technologies of the next decade are likely to bring innovations such as terahertz radars, which can operate in a higher range (100–300 GHz) than the 20–50 GHz bands, with significantly better angular resolution [61]. Advances in FMCW LiDAR, increased modulation frequency and the introduction of integrated photonic chips will result in lower costs and more compact designs [8]. Neuromorphic event cameras with microsecond response times will enable vehicles to react more quickly to sudden events [64]. In addition, quantum sensors and metamaterial antennas represent radically new directions for future sensors: these technologies are capable of detecting even a single photon, with extremely low noise and a wide dynamic range. However, research should not focus solely on hardware; artificial intelligence algorithms must also evolve to effectively integrate data from different modalities and create generalised models that are adaptive, explainable and resistant to data errors. Sensor fusion pipeline visualisation: The following figure shows an imagined sensor fusion process where signals from camera, LiDAR, radar, GNSS/IMU and V2X sensors are combined in a central perception engine. The diagram illustrates how different modalities contribute to the environmental model and how the system’s decision-making modules (planning, control) use this information for safe operation.

**Figure 9:**
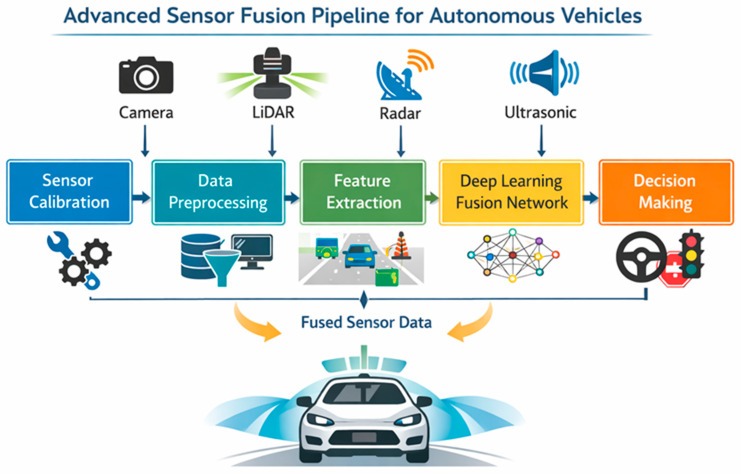
Sensor fusion process. Source: own edited.

Several publications deal with the details of fusion algorithms and systems: Rohling et al. [45] conducted a benchmark study on raw data fusion of sensors; Zang et al [4], Valverde et al. [26] and Vinoth and Sasikumar. [27] analysed the advantages of deep learning and Bayesian approaches. Weng and Zhang. [43] investigated the scalability of large-scale fusion systems, while Huang and colleges [52] proposed fuzzy logic methods. Antoniou et al. [55] presented generative methods for generating synthetic sensor data, Park and Ko [53] discussed the integration of radar micro-Doppler information, Kim and Ling [46] presented deep learning-supported FMCW radar processing, Gallego and colleges [47] discussed band- and feature-level camera-LiDAR fusion, Li et al. [66] presented radar-camera SLAM, and Paden and colleges [67] presented fusion algorithms for event cameras. The common lesson from these studies is that fusion architectures must be adaptive, learn in real time, and be synchronised for safe autonomous operation (Figure 9).

Human factors and social acceptance: The biggest challenge for autonomous vehicles is not necessarily technical but human and ethical. User confidence can only be gained gradually: the system must communicate transparently about the status of sensors, the uncertainty of algorithms, and the necessary human interventions. Cultural differences also play a role: while some countries are open to autonomous technologies, other regions are characterised by strong scepticism and regulatory caution. Consumer acceptance can be increased if the user experience of the vehicles is positive (comfortable, safe, energy-efficient) and if the system complies with personal data protection legislation. Ethical dilemmas and legal issues: Ethical dilemmas inevitably arise when programming autonomous vehicles: What decision should the vehicle make if all options involve injury? How much can we trust decisions made by artificial intelligence, and how can their appropriateness be verified? Current legal systems are mostly optimised for human drivers, and legislators urgently need to develop frameworks that address the liability of autonomous systems, the legal consequences of accidents, and insurance issues. According to the concept of algorithmic responsibility, developers and operators are responsible for the decisions made by the system; this requires transparent algorithms and auditable logic.

Global perspective and social impact: The autonomous vehicles of the future will be part of a global network. Technologies developed in countries in the northern hemisphere may not necessarily work effectively in the changed climatic and infrastructural conditions of the southern hemisphere. In developing countries, poor road surfaces, informal traffic rules and a lack of communication infrastructure all pose challenges. It is important that sensor systems are adaptable to different environments and that local needs are taken into account when transferring technology. The impact of autonomous vehicles on the labour market (e.g., taxi drivers, transporters), urban planning and the environment also needs to be considered; these are issues that researchers and policymakers need to address together.

A cautious path to development and the prospects for Level 5 autonomy: Although the media often touts the imminent arrival of fully autonomous driving (Level 5) without human intervention, in reality this is still decades away. Technological obstacles (perception, prediction, planning), legal and ethical issues, infrastructure development and social acceptance all take time. The likely scenario for the coming years is gradual autonomy: cars will perform more and more driving assistance functions (Level 2–3), and higher levels of autonomy (Level 4) will become available in special environments (motorways, industrial parks). To achieve this, developers need to design flexible and upgradeable platforms where software and hardware modules can be replaced over the years. Based on the above, it is clear that research into sensors for autonomous vehicles is a complex task that goes beyond the traditional boundaries of engineering. The benefits (safety, efficiency, accessibility) are significant but can only be realised if the technology is developed in a responsible, ethical and sustainable manner. The basis for future work is multidisciplinary collaboration: engineers, physicists, data scientists, ethicists, lawyers and social scientists are working together to develop systems that are not only technologically advanced but also socially useful. Only in this way can the goal be achieved that autonomous vehicles are not just technological marvels but the cornerstones of sustainable, safe and fair transport. These ideas are in line with the latest literature, which emphasises the need to develop legal and ethical frameworks for autonomous vehicles [59,60], emphasises the importance of interior passenger monitoring and passenger safety [65], and highlights the role of adaptive, weather-sensing management in system reliability [66].

## 5. Conclusions

Based on the analysis presented in this review, several key technological and research directions can be identified for the future development of autonomous vehicle sensing systems. The sensor systems of autonomous vehicles are one of the most critical and rapidly developing components of intelligent transport systems. The aim of this review was to provide a comprehensive, systematic and critical overview of the current state of sensor technologies used in self-driving vehicles, their advantages, limitations and future development directions. Based on a systematic review of the literature, it has become clear that the problem of autonomous sensing goes far beyond the technical parameters of individual sensors: in fact, it is a question of a complex, multi-level integrated system in which hardware, software, safety, cost and environmental robustness are closely intertwined.

One of the central findings of the article is that there is no universally “best” sensor technology for autonomous vehicles. Each type of sensor, whether camera, LiDAR, radar, ultrasound or localisation sensors, has its own well-defined strengths and weaknesses. Cameras provide outstanding semantic information but are sensitive to lighting conditions and weather. LiDAR enables extremely accurate spatial mapping but is expensive and sensitive to environmental influences. Radar is robust in bad weather, but its resolution is limited. Ultrasound is effective for close-range detection, but its range is limited. GNSS and IMU-based localisation is essential for global positioning but is not sufficient on its own in dynamic environments.

It follows that the future of autonomous vehicles will necessarily be based on multi-sensor architectures. Complementarity between sensors is not optional, but a fundamental design principle. Redundancy, diverse sensing principles and multi-source information fusion together can provide the robustness that is essential in real-world traffic environments. Autonomous driving is therefore not about the “victory” of a single sensor but about the cooperation of heterogeneous systems. This review has also highlighted that environmental robustness remains one of the biggest open questions. Most publications present results under ideal or near-ideal conditions, while real traffic situations often involve adverse weather, contaminated sensors, extreme lighting conditions and complex dynamic situations. Under such conditions, sensor performance often does not deteriorate in a binary manner but gradually degrades, which is particularly dangerous because it is more difficult to detect. This phenomenon highlights the importance of uncertainty modelling and reliability estimation.

One important conclusion of the article is that future autonomous sensor systems must not only detect objects but also quantify their own uncertainty. Probabilistic perception, uncertainty-aware neural networks and Bayes-based fusion methods are expected to play an increasingly important role. The safety of autonomous decision-making depends largely on the system’s ability to recognise its own limitations. The dominance of data-driven approaches has also brought new types of challenges. The performance of deep learning models depends heavily on training data, which is often biassed in terms of geography, culture and weather. The underrepresentation of “long-tail” events and rare but critical situations poses a serious safety risk. In the future, it will be essential to collect diverse, global data sets and to make conscious use of simulation and synthetic data generation. With regard to sensor fusion, the article concludes that, although it has enormous potential, it cannot be considered a miracle solution. Calibration, time synchronisation and the alignment of coordinate systems remain critical technical challenges. Learned fusion models are promising, but their validation and certifiability are still immature. In the future, great emphasis must be placed on the formal verification and safety assessment of fusion systems. The dimension of functional safety is particularly important. The ISO 26262 and ASIL requirements represent not only a regulatory framework but also a design philosophy. True safety redundancy requires diverse redundancy, i.e., a combination of sensors operating on different physical principles. Diagnostics, self-monitoring and degraded mode management will be key in future autonomous systems.

Emerging technologies such as FMCW LiDAR, terahertz radar and neuromorphic cameras have significant potential, but their industrial maturity is still variable. It is likely that in the short to medium term, these will play a complementary role rather than resulting in a complete technology shift. Cost, integrability and standardisation remain key factors.

This study also pointed out that energy consumption and system architecture are increasingly important design considerations. Computationally intensive sensing involves significant heat generation and energy consumption, which can limit scalability. Energy-efficient sensors, neural accelerators and edge-based processing are therefore of strategic importance. The social and ethical dimensions cannot be ignored either. Cameras and other sensors raise privacy concerns, particularly due to the recording of personally identifiable information. Privacy-by-design approaches, anonymisation and edge processing can play an important role in increasing social acceptance.

The key message of the article is that autonomous vehicle perception is a system-level problem. Future research should focus not only on the development of individual sensors but also on integration, validation, uncertainty management and safety. Technological progress alone is not enough; reliable engineering methodologies, standards and validation frameworks are also needed.

Future autonomous vehicles are expected to use sensor systems that

- Have a diverse set of sensors,
- Explicitly handle uncertainty,
- Are based on energy-efficient architecture,
- Meet strict functional safety requirements,
- Are capable of adapting to the diversity of the global environment.

Ultimately, the perception problem of autonomous driving is not just an engineering issue but an interdisciplinary challenge involving artificial intelligence, physical perception, system safety, ethics and regulation. Research over the next decade will play a decisive role in determining the extent to which autonomous vehicles become a truly safe, reliable and widely accepted transport solution.

## Figures and Tables

**Table 1:** SAE levels of driving automation based on SAE J3016. Source: [2] own edited.

SAE Level	Description	Driver Responsibility
Level 0	No automation	Human driver performs all driving tasks
Level 1	Driver assistance	System assists with steering or acceleration
Level 2	Partial automation	System controls steering and speed but driver must supervise
Level 3	Conditional automation	System performs driving tasks in specific environments
Level 4	High automation	Vehicle can operate autonomously in defined areas
Level 5	Full automation	Vehicle performs all driving tasks in all conditions

**Table 2:** Typical characteristics of visual sensors used in autonomous vehicles based on values reported in the literature [24,25,26,27,30,39].

Sensor Type	Typical Detection Range	Dynamic Range	Key Advantages	Main Limitations
RGB camera [23]	100–200 m	~60 dB	High spatial resolution, colour and texture information, low cost, and wide availability	Strong sensitivity to lighting conditions, glare and shadows; no direct depth measurement
HDR camera [24]	100–200 m	90–100 dB	Improved visibility in high contrast scenes, reduced overexposure	Higher computational requirements, possible ghosting in dynamic scenes
Event camera [25,26,27,28,29]	30–100 m	120–140 dB	Extremely high temporal resolution (microseconds), no motion blur, efficient data generation	Lower spatial resolution, requires specialised algorithms and event-based processing
Thermal camera (LWIR) [36,37,38]	up to 200 m (pedestrians)	~80 dB	Works in darkness, detects heat-emitting objects such as pedestrians or animals	Higher price, lower spatial resolution, limited texture information

**Table 3:** Comparison of distance sensors. Source: own edited.

Sensor	Range	Point Resolution	Speed Detection	Weather Sensitivity	Note
**ToF LiDAR (mechanical/solid-state)**	150–300 m	Centimetre	Indirect (by comparing point clouds over time)	High: rain and fog weaken signal reflection [2]. Maintenance requirements	High for rotating parts
**FMCW LiDAR**	200–300 m	Centimetres	Direct: based on Doppler frequency [8] signal-to-noise ratio	Medium: coherent detection improves CMOS [9]	High cost, but can be integrated
**Millimetre-wave radar (24–81 GHz)**	50–250 m	Ten centimetres	Direct speed measurement (Doppler)	Low: works well in rain and fog [6]	Low resolution, small size
**Terahertz radar (0.3–3 THz)**	100–300 m	~centimetre	Direct speed measurement	Low: good in fog and rain [9] resolution	Experimental stage, more expensive but better
**Ultrasonic sensor**	0–5 m	Several centimetres	Limited	Moderate: sensitive to temperature and wind [6]. Widespread	Inexpensive, used in parking systems

**Table 4:** Typical atmospheric attenuation of LiDAR wavelengths under different weather conditions. Source: compiled from studies on atmospheric attenuation of automotive LiDAR signals [3,15,16].

Weather Condition	Attenuation at 905 nm	Attenuation at 1550 nm	Remarks
Clear air	<0.01 dB/km	<0.01 dB/km	Negligible atmospheric absorption
Light rain (2 mm/h)	0.1–0.3 dB/km	0.05–0.2 dB/km	Minor signal loss
Heavy rain (25 mm/h)	1–2 dB/km	0.5–1.5 dB/km	Reduced detection range
Light fog (visibility ~500 m)	5–10 dB/km	3–7 dB/km	Significant scattering
Dense fog (visibility < 50 m)	50–100 dB/km	30–70 dB/km	Severe attenuation
Snowfall	10–30 dB/km	5–20 dB/km	Strong scattering depending on flake size

**Table 5:** Representative LiDAR detection range under different precipitation intensities. Source: own edited.

Weather Condition	Attenuation at 905 nm	Attenuation at 1550 nm	Remarks
Clear air	<0.01 dB/km	<0.01 dB/km	Negligible atmospheric absorption
Light rain (2 mm/h)	0.1–0.3 dB/km	0.05–0.2 dB/km	Minor signal loss
Heavy rain (25 mm/h)	1–2 dB/km	0.5–1.5 dB/km	Reduced detection range
Light fog (~500 m visibility)	5–10 dB/km	3–7 dB/km	Significant scattering
Dense fog (<50 m visibility)	50–100 dB/km	30–70 dB/km	Severe attenuation
Snowfall	10–30 dB/km	5–20 dB/km	Strong scattering depending on flake size

**Table 6:** Representative sensor fusion architectures used in autonomous vehicle perception systems. Source: compiled from reported results on KITTI and nuScenes benchmarks [37,46,49,50].

Method	Fusion Level	Input Modalities	Data Set/Benchmark	Reported Performance	Computational Requirements
MV3D	Feature-level	LiDAR + camera	KITTI	~74% mAP (3D detection)	High GPU load
PointPainting	Early fusion	LiDAR + camera	KITTI	~77–80% mAP	High memory bandwidth
BEVFusion	Mid-level	LiDAR + camera	nuScenes	~71–74% mAP	High GPU requirement
BEVFormer	Transformer fusion	Multi-camera (+ optional LiDAR)	nuScenes	~56–60% NDS	Very high compute
CenterFusion	Feature-level	Radar + camera	nuScenes	~52% NDS	Moderate–high
DeepFusion	Multi-modal	Camera + LiDAR + radar	nuScenes	~75% NDS	Very high

**Table 7:** Examples of autonomous vehicle sensing platforms. Source: own edited.

Platform	Developer	Main Sensors	Typical Detection Range	Vehicle Range
Waymo robotaxi	Waymo (Alphabet)	LiDAR, cameras, radar	LiDAR up to ~300 m	~400 km
Tesla FSD platform	Tesla	8 cameras, radar, ultrasonic sensors	Cameras up to ~250 m	400–600 km
Baidu Apollo	Baidu	LiDAR, cameras, radar	LiDAR ~200–250 m	~400 km
Cruise AV	General Motors	LiDAR, radar, cameras	LiDAR ~250 m	~350 km

**Table 8:** Comparison of safety characteristics. Source: own edited.

Aspect	Human-Driven Vehicles	Autonomous Vehicles
Reaction time	~1–2 s	milliseconds
Fatigue/distraction	frequent	none
Perception range	limited by human vision	extended by sensors
Failure modes	human error	sensor/software failure
Redundancy	none	multi-sensor fusion

**Table 9:** Effects of lightning strikes on sensors. Source: own edited.

Weather Phenomenon	Camera	LiDAR	Radar	Ultrasound	Comment
**Fog/humid air**	Significantly reduces contrast, high noise	High extinction due to Mie scattering, reduced range [11], meg [11] droplets	Slight reduction in signal-to-noise ratio; rain noise may occur	Minor effect, but fog-like	Thermal cameras and terahertz radars work better in fog
**Rain**	Lens fogging, contrast reduction	Range decreases, signal intensity deteriorates	Background noise caused by raindrops, false alarms	Sound waves are slightly reduced by water droplets	In heavy rain, windscreen wipers can also impair GNSS signal strength [11]
**Snow**	Covers the lens, reflects light	Moderate range reduction, diffuse scattering	Snow crystals have relatively little disruptive effect	Sound absorption increases	Heated sensors reduce snow accumulation
**Glare from sunlight**	Over-exposes the image, HDR camera required	No effect, as LiDAR uses its own light	No effect	No effect	Camera systems can be improved with polarised filters

## Data Availability

No new data were created or analyzed in this study.

## Abbreviations

GNSS	Global Navigation Satellite System
GPS	Global Positioning System
IMU	Inertial Measurement Unit
LiDAR	Light Detection and Ranging
RADAR	Radio Detection and Ranging
EKF	Extended Kalman Filter
UKF	Unscented Kalman Filter
AV	Autonomous Vehicle
ADAS	Advanced Driver Assistance Systems
INS	Inertial Navigation System
